# Unloading Mechanical Behaviors of Clay Considering Initial Principal Stress Direction Effect: Experimental Study and Modified Constitutive Model

**DOI:** 10.3390/ma19173581

**Published:** 2026-08-24

**Authors:** Ping Hu, Wei-Xun Zhang, Yi Han, Xiang-Hua Song, Le Zhang, Jia-Yu Ruan, Jun-Cheng Liu

**Affiliations:** 1 School of Civil Engineering and Architecture, University of Jinan, 336 West Nanxinzhuang Road, Jinan 250022, China; 2 College of Civil Engineering, Fuzhou University, Fuzhou 350108, China

**Keywords:** depositional orientation, triaxial unloading test, Duncan–Chang model, tangent modulus, clay

## Abstract

Excavation-induced unloading can alter soil response through both stress-path effects and material orientation. This study investigates remolded clay from Jinan using conventional consolidated undrained (CCU) and lateral unloading undrained (LUU) triaxial tests at orientation angles α = 0°, 30°, 45°, 60°, and 90° and effective consolidation pressures of 100 and 200 kPa. The measured responses were strain-hardening, with no objective yield point or distinct peak. Under CCU, the operational failure stress generally decreased from α = 0° to 60° and increased slightly at 90°, while the fitted initial tangent modulus decreased with α and the hyperbolic asymptotic deviator stress was highest at 45°. Under LUU, the terminal deviator stress showed pressure-dependent orientation effects, and the initial tangent modulus decreased with α. The apparent cohesion was lowest at 45°, whereas the apparent friction angle was lowest at 90°. An orientation-dependent Duncan–Chang extension was developed for the prescribed LUU path by parameterizing *K*, *n*, *R_f_*, *c*, and *φ* as functions of α. An internal hold-out check at α = 60° gave parameter deviations of 2.1% or less, supporting interpolation within the investigated orientation range. Accordingly, the formulation is interpreted as an empirical fixed-orientation relation for the investigated LUU path.

## 1. Introduction

Supported excavations for basements, metro stations, and other underground structures can induce substantial stress relief and redistribution in the surrounding ground, potentially affecting adjacent buildings and infrastructure [1,2,3]. During excavation, the progressive release of lateral stresses induces stress redistribution in the surrounding ground and can lead to pronounced deformation of the soil and adjacent underground structures [4,5,6]. Soil parameters used in routine geotechnical analyses are commonly obtained from conventional triaxial compression tests. However, field observations and excavation analyses have shown that soils adjacent to supported excavations may follow stress paths involving substantial lateral stress relief, which differ from conventional compression paths [7]. Consequently, parameters derived solely from conventional compression tests may not fully represent the path-dependent stiffness and mobilized resistance under excavation-related unloading conditions. In addition to the unloading stress path itself, changes in principal-stress direction can further influence the mechanical response of geomaterials [8,9,10]. These combined stress-path and orientation effects therefore need to be considered when evaluating soil behavior under excavation-related unloading conditions.

To investigate the mechanical behavior of geomaterials subjected to unloading stress paths, numerous experimental studies have been conducted worldwide. Zeng et al. [11] performed cyclic triaxial tests on silica powder and Fujian sand to investigate unloading elastic behavior and demonstrated that the unloading tangent Young’s modulus is primarily controlled by stress state and porosity, while being independent of cyclic amplitude. They further revealed that the unloading modulus exhibits greater sensitivity to axial stress than to lateral stress, and that the unloading average Poisson’s ratio is also closely related to stress state and porosity. Lu et al. [12] proposed a power-function-based model incorporating the initial modulus and decay rate to describe the evolution of the one-dimensional unloading modulus with unloading ratio. Their results indicated that the initial unloading modulus increases linearly with effective vertical stress prior to unloading, and this relationship can also be represented using a power function. Shu et al. [13] reported that unloading significantly accelerates the accumulation of axial plastic strain, with the effect becoming more pronounced as the unloading magnitude increases. Compared with other initial stress paths, soils subjected to unloading paths exhibit reduced cyclic resistance. Liu et al. [14] conducted cyclic triaxial tests on Sydney sand samples of varying relative densities, with staged unloading of confining pressure applied between loading cycles, to investigate the effect of unloading history on subsequent cyclic deformation. Their results showed that cumulative strain increases with unloading amplitude due to the unloading memory effect, whereas the strain development direction is controlled only by the mean stress ratio and is independent of soil density and stress path. Liu et al. [15] conducted undrained cyclic triaxial tests on kaolin clay to explore the influence of unloading on subsequent cyclic mechanical behavior. They found that increasing unloading amplitude accelerates the accumulation of cyclic strain and excess pore water pressure, while the strain evolution direction is governed by the shear stress ratio. Furthermore, the unloading procedure has little influence on the final cumulative strain, and the acceleration of deformation is primarily attributed to changes in stress state and unloading history effects. Liu et al. [16] carried out seepage–stress coupled triaxial confining-pressure unloading tests on sandstone using a multi-channel experimental system. Their findings indicated that unloading induces a transition in failure mode from predominantly shear failure to combined tensile–shear failure. Additionally, the peak permeability occurs after the acoustic emission peak, and the discrepancy between permeability evolution and failure behavior is controlled by the deviator stress level. More directly related to excavation-induced stress relief and lateral ground movement, Ng [17] investigated field effective-stress paths adjacent to a diaphragm wall during excavation and compared them with laboratory triaxial stress paths representing horizontal and vertical stress relief. Hashash and Whittle [18] further examined the evolution and redistribution of stresses around braced excavations in clay, highlighting the importance of stress-path changes and stress reversals during excavation. Finno and Cho [19] conducted stress-probe tests on high-quality clay samples subjected to different recent stress histories, including an unloading path representative of urban excavation conditions, and demonstrated a pronounced influence of the unloading history on soil stiffness response. These studies provide direct field, analytical, and experimental evidence that excavation-induced stress relief and lateral soil movement are associated with stress-path-dependent mechanical responses, providing relevant background for the prescribed lateral unloading path investigated in the present study.

The initial principal stress direction represents a critical factor influencing the accuracy of soil constitutive modeling under unloading conditions. The Duncan–Chang model and its variants [20,21,22,23] have been widely used to describe nonlinear stress–strain behavior in soils and other geomaterials, with model parameters commonly calibrated from triaxial test data. Consequently, this model exhibits limited capability in describing stress path-dependent behavior involving principal stress rotation and lacks an explicit criterion for distinguishing loading and unloading processes. These limitations hinder its applicability to the complex stress conditions associated with supported excavations, where principal stress rotation and stress redistribution may occur. The cited recent Duncan–Chang-based applications and adaptations mainly account for material- or condition-dependent effects through parameter calibration or correction, whereas the specific combination of a prescribed lateral unloading undrained path and fixed material orientation relative to the applied principal-stress axes has received limited explicit treatment in the cited Duncan–Chang-based literature. The present extension addresses this gap in two linked respects: the tangent-modulus formulation is specialized to the axisymmetric LUU path, and the orientation effect is introduced explicitly through *K*(α), *n*(α), *R_f_*(α), *c*(α), and *φ*(α). Accordingly, its distinguishing feature is the joint path-specific and orientation-conditioned representation of the unloading response within the Duncan–Chang hyperbolic framework. Therefore, systematic investigation of soil unloading deformation considering different initial principal stress directions, together with the development of an excavation unloading constitutive model incorporating principal stress direction effects, is of significant theoretical and practical importance. Such efforts can contribute to a more accurate understanding of soil stress responses during excavation, improve the theoretical framework of foundation pit design, and enhance engineering prediction capabilities. Therefore, this study investigates the effects of initial material orientation on the mechanical behavior of clay under lateral unloading conditions, focusing on stress–strain response, strength characteristics, and deformation modulus evolution under different stress paths. A controlled experimental framework was established by preparing clay specimens with different orientation angles relative to the deposition direction, allowing the coupled effects of material orientation and unloading stress path to be systematically evaluated. Building on the conventional Duncan–Chang framework, an orientation-dependent LUU extension was developed in which the parameters *K*, *n*, *R_f_*, *c*, and *φ* were related to the orientation angle α and incorporated into the tangent-modulus formulation. A series of triaxial compression and lateral unloading tests were conducted under different initial principal stress orientations and consolidation pressures to characterize the anisotropic mechanical response of clay. The resulting formulation provides a material-level framework for describing the tested fixed-orientation LUU response and for subsequent evaluation in supported-excavation analyses.

## 2. Materials and Methods

The clay was obtained from the Jinan Yellow River Tunnel construction area in China. The available index properties are summarized in Table 1. The water content and bulk density were 28.0% and 1.79 g/cm^3^, respectively, while the liquid and plastic limits were 41.8% and 25.9%, respectively. The degree of saturation was not directly determined as an initial index property; instead, specimen saturation prior to consolidation and shearing was verified using the Skempton B-value criterion, as described below.

The remolded clay blocks were prepared using a pressure-assisted slurry consolidation method. The collected soil was first air-dried, gently crushed, and passed through a 0.3 mm stainless-steel sieve. Distilled water was then gradually added, and the mixture was mechanically stirred for approximately 5–10 min until a homogeneous and flowable slurry without visible aggregates was obtained. The slurry was slowly poured along the inner wall of the consolidation chamber and allowed to stand for approximately 12 h to facilitate the release of entrapped air.

**Figure 1:**
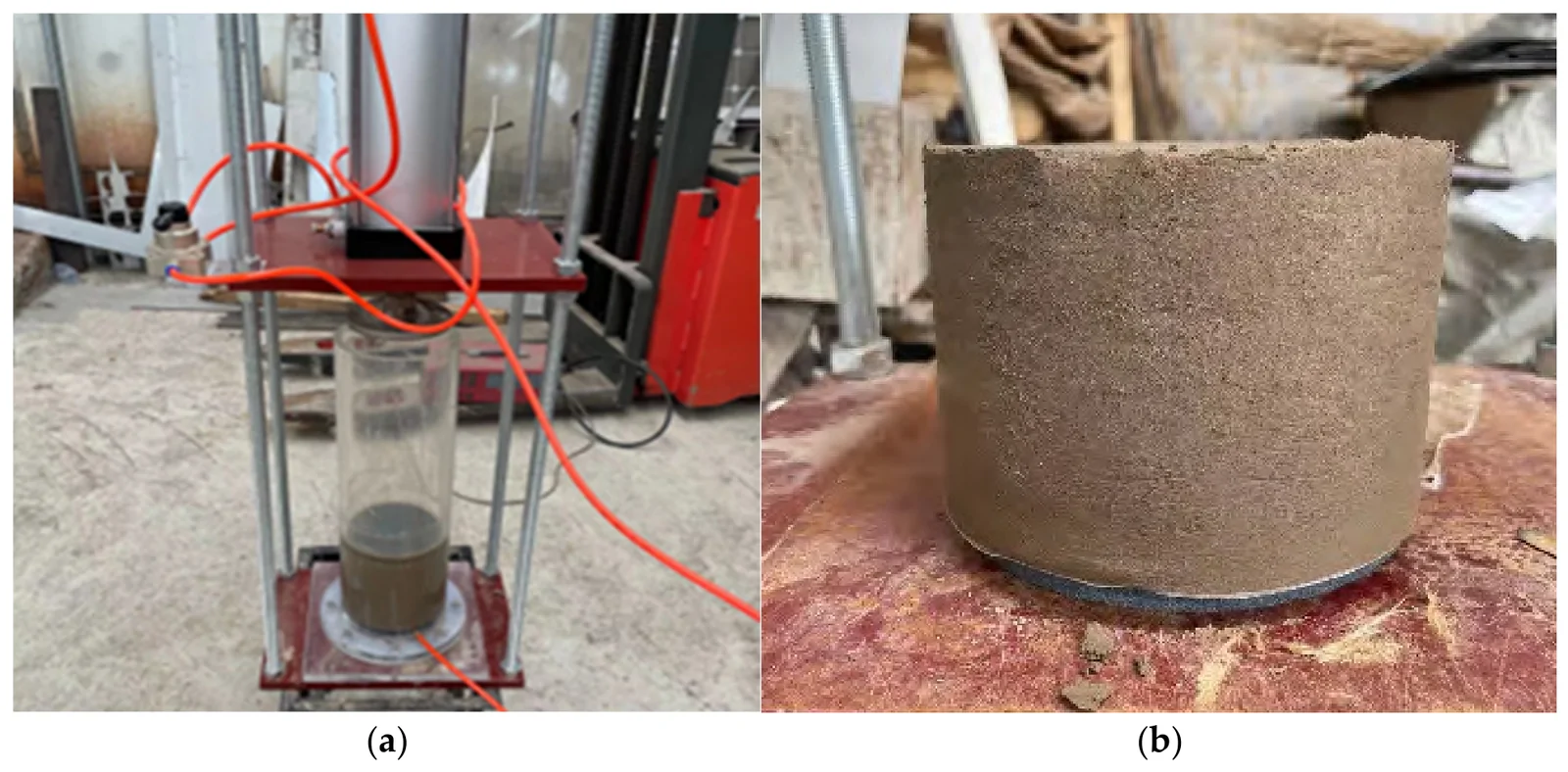
Reshaped clay sample. (**a**) Slurry consolidation loading. (**b**) Reshaped clay sample.

A self-developed pneumatic consolidation apparatus was subsequently used to consolidate the slurry, as shown in Figure 1. The cylindrical consolidation chamber had an inner diameter of 150 mm, a height of 500 mm, and a wall thickness of 10 mm. Filter paper and porous stones were placed at the top and bottom of the soil slurry, and a drainage outlet was provided at the base of the chamber. Pneumatic pressure was applied in three stages to a final consolidation pressure of 100 kPa. At each stage, the next pressure increment was applied after drainage from the bottom outlet had essentially ceased. After completion of consolidation, the resulting clay block was carefully removed from the chamber for subsequent directional sampling.

**Figure 2:**
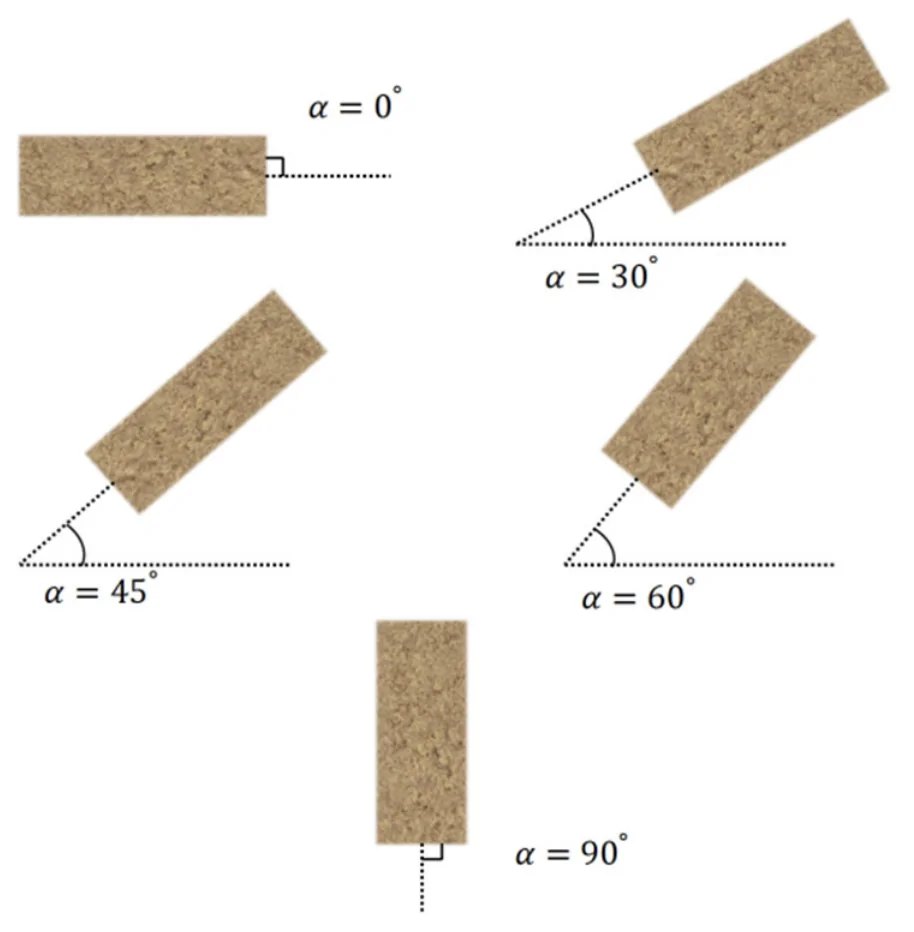
Schematic diagram of specimen sampling directions relative to the deposition direction.

After pressure consolidation, the clay blocks were sampled along predetermined orientations of 0°, 30°, 45°, 60°, and 90° relative to the deposition direction. The directionally sampled blocks were carefully trimmed into cylindrical triaxial specimens with a diameter of 39.1 mm and a height of 80 mm. No kneading, reconstruction, or additional remolding was performed after directional sampling. The specimen dimensions, water content, and bulk density were checked before testing to ensure consistency among the prepared specimens. The directional sampling procedure is illustrated in Figure 2. No direct microstructural characterization was performed to verify the deposition-induced fabric or its preservation after specimen preparation. Accordingly, references to fabric orientation, particle arrangement, and structural anisotropy in the following discussion are presented only as possible interpretations of the macroscopic test results, rather than as directly verified mechanisms. The orientation angle α is defined as the angle between the specimen axial direction and the deposition direction of the consolidated clay block.

**Figure 3:**
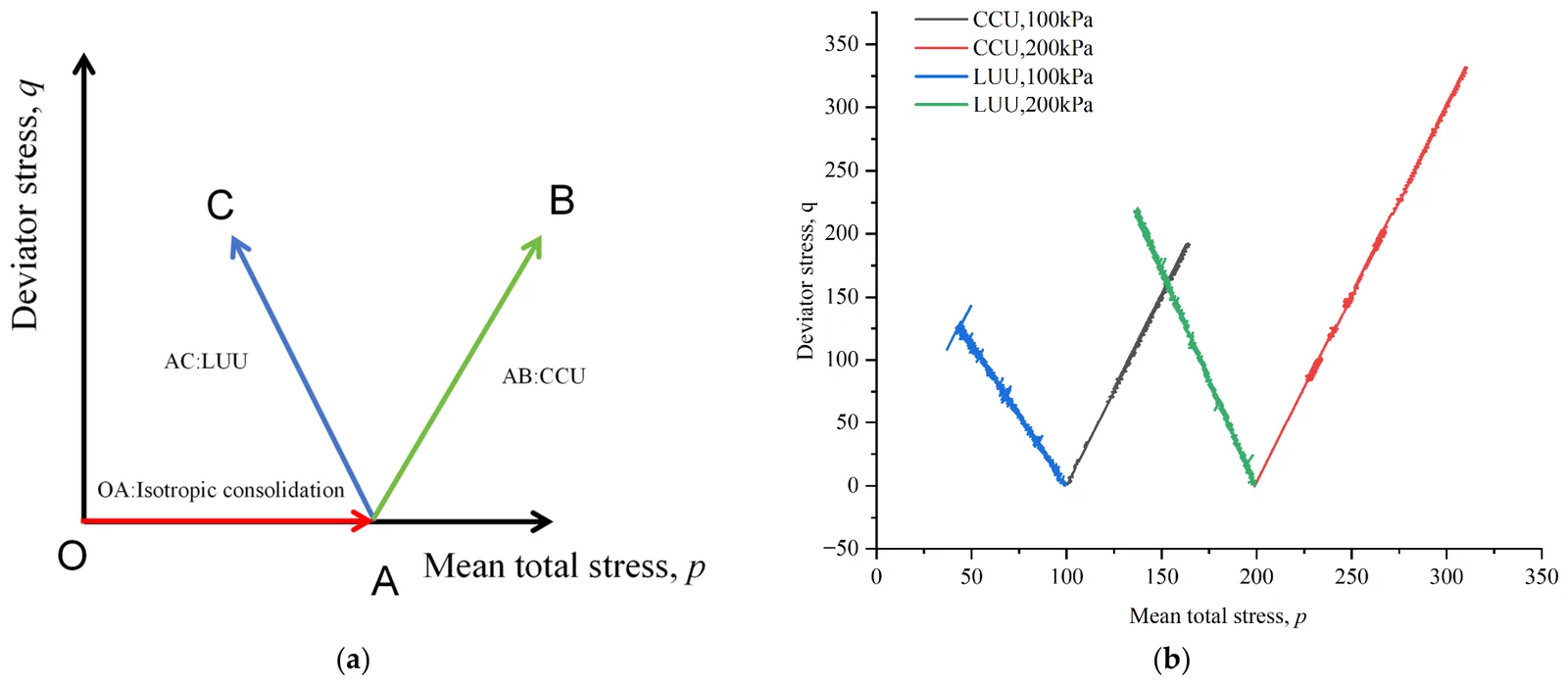
Stress paths in *p*-*q* space: (**a**) idealized stress paths for the CCU and LUU tests; (**b**) representative measured total-stress paths for α = 0° at consolidation pressures of 100 and 200 kPa.

The mechanical tests were performed using a high-precision servo-controlled stress-path triaxial testing system (Nanjing, China). Two representative stress-path conditions were adopted in the experimental program: (1) lateral unloading undrained shear (LUU) tests, in which the axial stress was maintained constant while the lateral confining pressure was gradually reduced, thereby representing the lateral stress-release path experienced by soil adjacent to a supported excavation; and (2) conventional consolidated undrained (CCU) compression tests, which were conducted as a reference condition to evaluate the differences in soil mechanical behavior between unloading and conventional loading stress paths. The schematic and representative measured stress paths are presented in Figure 3. Figure 3a illustrates the prescribed CCU and LUU stress paths [24], where path OA represents isotropic consolidation, path AB denotes conventional consolidated undrained compression, and path AC represents lateral unloading undrained shearing. Figure 3b further presents representative measured total-stress paths for α = 0° at consolidation pressures of 100 and 200 kPa.

Before consolidation, the triaxial specimens were saturated using the vacuum saturation method. The specimens were placed in a vacuum saturation apparatus, where air was evacuated before water was gradually introduced. Saturation was maintained for at least 24 h. After specimen installation in the triaxial apparatus, the pore-pressure coefficient B was measured to verify saturation, and only specimens with B ≥ 0.95 were subjected to subsequent isotropic consolidation at effective confining pressures of 100 or 200 kPa. Considering the rapid excavation and support characteristics commonly adopted in foundation pit construction, together with the low permeability of saturated clay, all shear tests were conducted under undrained conditions. All tests were performed in accordance with the Standard for Geotechnical Testing Method (GB/T 50123–2019) [25]. The orientation angles α = 0°, 30°, 45°, 60°, and 90° were selected to cover the investigated range between the two limiting orthogonal orientations, with intermediate angles included to capture the orientation-dependent variation in mechanical response. Effective consolidation pressures of 100 and 200 kPa were adopted as two distinct confinement levels to examine the influence of stress level on the orientation-dependent response. The testing program is summarized in Table 2. During the CCU shearing stage, pore-water pressure was continuously monitored. For the representative records reported below, the excess pore-water pressure, Δu, is expressed relative to the pore-water pressure at the beginning of undrained shearing.

One specimen was tested for each combination of orientation angle, consolidation confining pressure, and stress path, resulting in a total of 20 triaxial tests. Replicate triaxial tests were not conducted for individual test conditions; therefore, standard deviations or error bars are not available for the mechanical responses reported herein. This limitation should be considered when interpreting relatively small differences among individual test curves, and the present results are primarily used to identify the overall orientation- and stress-path-dependent trends of the tested clay.

Because the measured stress–strain curves exhibited strain-hardening behavior and no objective yield-point identification procedure was applied in the present study, yield stress and yield strain are not reported. For the CCU tests without a distinct stress peak, the operational failure stress was taken as the representative deviator stress within the 15–20% axial-strain range, following the original test protocol. For the LUU tests, no distinct peak was observed within the recorded unloading range; therefore, the stress recorded at the end of the prescribed unloading path is referred to as the terminal deviator stress rather than yield or peak stress. In addition, the ultimate deviator stress obtained from hyperbolic fitting, $q_{ult}=1/b$, represents a theoretical asymptotic stress and is distinguished from the directly measured stress quantities throughout this paper.

## 3. Results

### 3.1. Analysis of Stress–Strain Relationship

**Figure 4:**
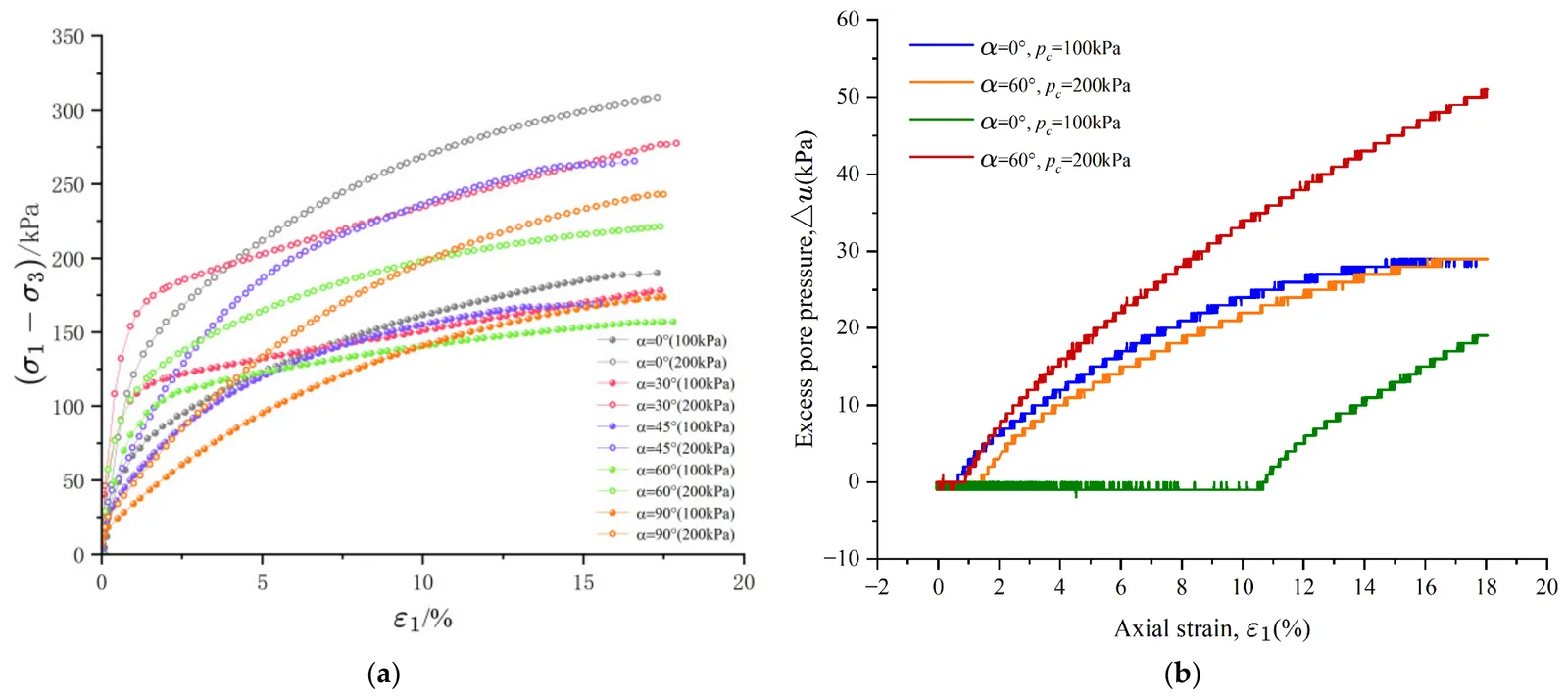
CCU responses. (**a**) Stress–strain curves. (**b**) Representative excess pore-water pressure–axial strain responses for α = 0° and 60° at consolidation pressures of 100 and 200 kPa.

**Figure 5:**
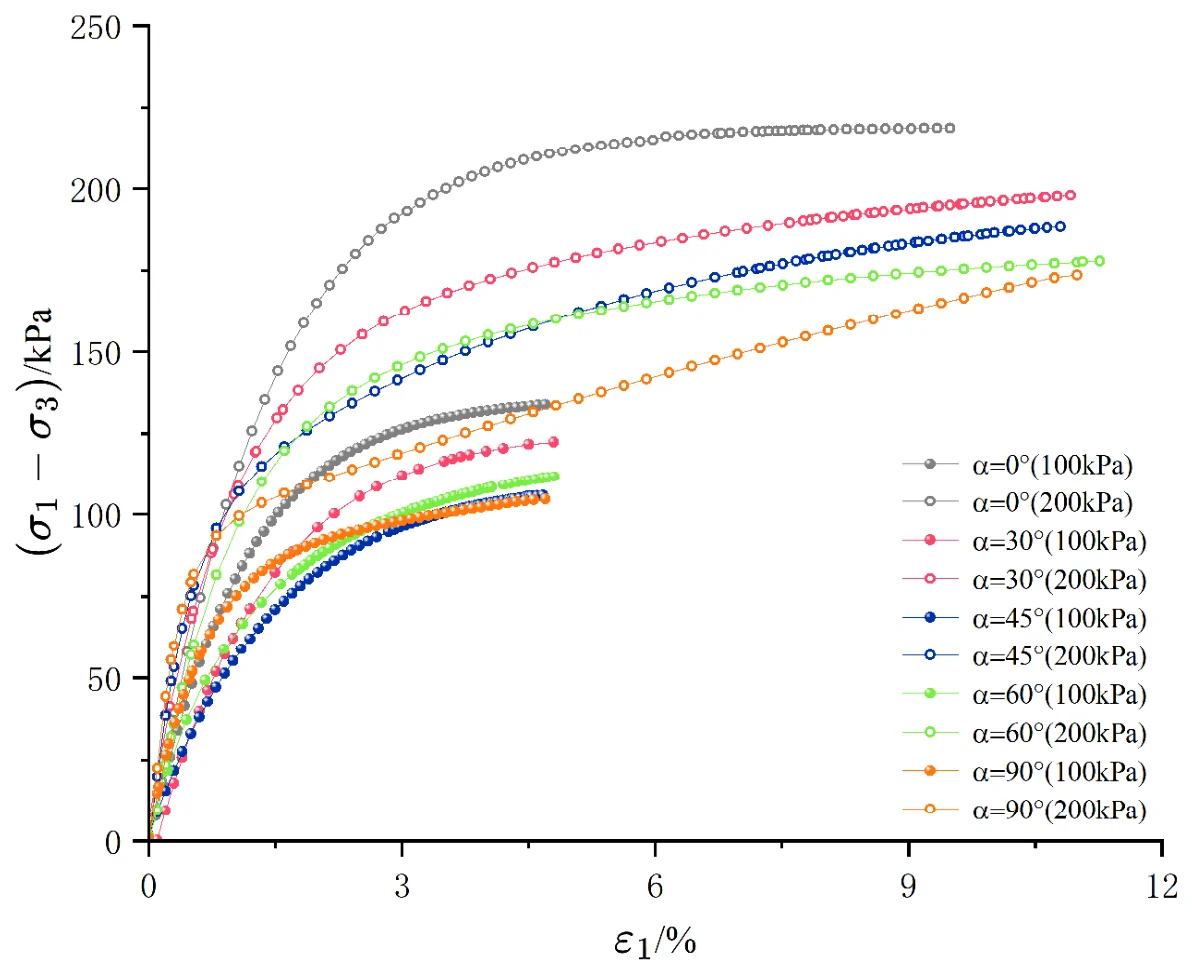
Stress–strain curves for the LUU tests.

Figure 4 and Figure 5 illustrate the stress–strain curves obtained from the CCU and LUU tests, respectively, under different initial principal stress direction angles (α) and consolidation confining pressures. A comprehensive examination of the test results indicates that, irrespective of variations in α and confining pressure, the stress–strain responses under both stress paths exhibit pronounced strain-hardening behavior. Specifically, the deviator stress continuously increases with increasing axial strain, without exhibiting an apparent post-peak strain-softening stage. This feature suggests that the stress–strain behavior of the tested clay can be effectively described using a hyperbolic constitutive relationship. A comparison between the two stress paths reveals distinct differences in their stress–strain responses. The initial portions of the curves obtained from the CCU tests (Figure 4) exhibit relatively gradual slopes, whereas those from the LUU tests (Figure 5) show steeper initial slopes, particularly at α = 0° and 30°. Thus, under the present testing protocols, the LUU tests exhibit a higher apparent initial stiffness than the CCU tests. However, because the two stress paths were conducted using different control modes and loading rates, this difference cannot be attributed solely to the unloading stress path. Although the initial portions of the curves are approximately linear, no objective yield-point identification procedure was applied; therefore, a specific yield stress or yield strain is not assigned.

In addition to the stress–strain responses, Figure 4b presents representative excess pore-water pressure–axial strain relationships obtained from the CCU tests at α = 0° and 60° under consolidation pressures of 100 and 200 kPa. These records show the measured pore-pressure evolution during the undrained shearing stage and are included to document the pore-pressure response corresponding to the CCU tests.

As axial strain increases, the tangent slope progressively decreases while the deviator stress generally continues to rise, consistent with the strain-hardening response observed over the recorded range. In this stage, the reduced rate of deviator-stress increase is consistent with progressive plastic deformation and a gradual mobilization of shear resistance before the adopted failure state is reached. Possible particle rearrangement or structural adjustment may contribute to this response.

For both test protocols, the characteristic stress levels increase with increasing consolidation pressure. Specifically, the operational failure stress adopted for the CCU tests and the measured terminal deviator stress of the LUU tests are higher at 200 kPa than at 100 kPa. At corresponding pressures, the CCU operational failure stress is generally higher than the LUU terminal deviator stress. This difference is consistent with the distinct prescribed stress evolutions of the two test protocols. Under the CCU path, axial shearing proceeds while lateral confinement is maintained, whereas the LUU path involves a progressive reduction in lateral confinement. Accordingly, the differences in the measured characteristic stress levels are described here as stress-path-dependent macroscopic responses.

The effect of consolidation pressure on the measured axial strain differs between the two test protocols. For the LUU tests, the axial strain recorded at the prescribed unloading endpoint ranged from approximately 3.5–5.0% at 100 kPa and 9.4–11.2% at 200 kPa. Thus, the endpoint axial strain was approximately twofold overall at the higher consolidation pressure, although the exact ratio depended on orientation. For the CCU tests, the differences in axial strain associated with consolidation pressure were comparatively less pronounced over the recorded strain range. This difference is observed under the prescribed LUU path, in which lateral confinement is progressively reduced as the stress state evolves during unloading.

Furthermore, the initial slopes of the stress–strain curves indicate that the LUU specimens generally exhibit a higher initial deformation modulus than the CCU specimens. This phenomenon may be related to the stress-dependent stiffness evolution of soil under unloading conditions. During lateral unloading, the gradual release of confining pressure changes the prescribed stress state and may contribute to the observed apparent stiffness response; however, particle-contact adjustment was not directly measured in this study.

**Figure 6:**
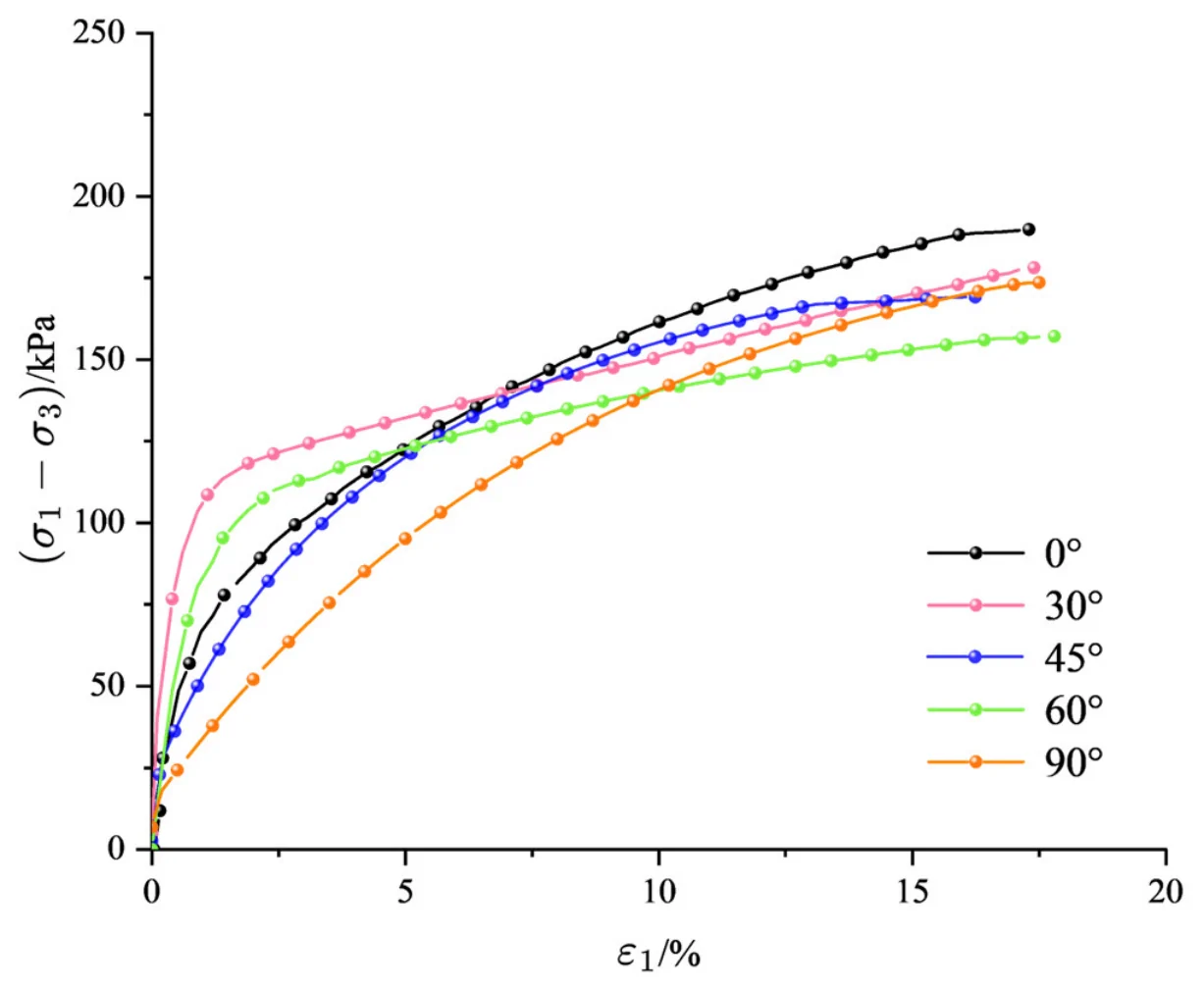
Stress−strain relationship diagram of CCU at consolidation confining pressure of 100 kPa.

**Figure 7:**
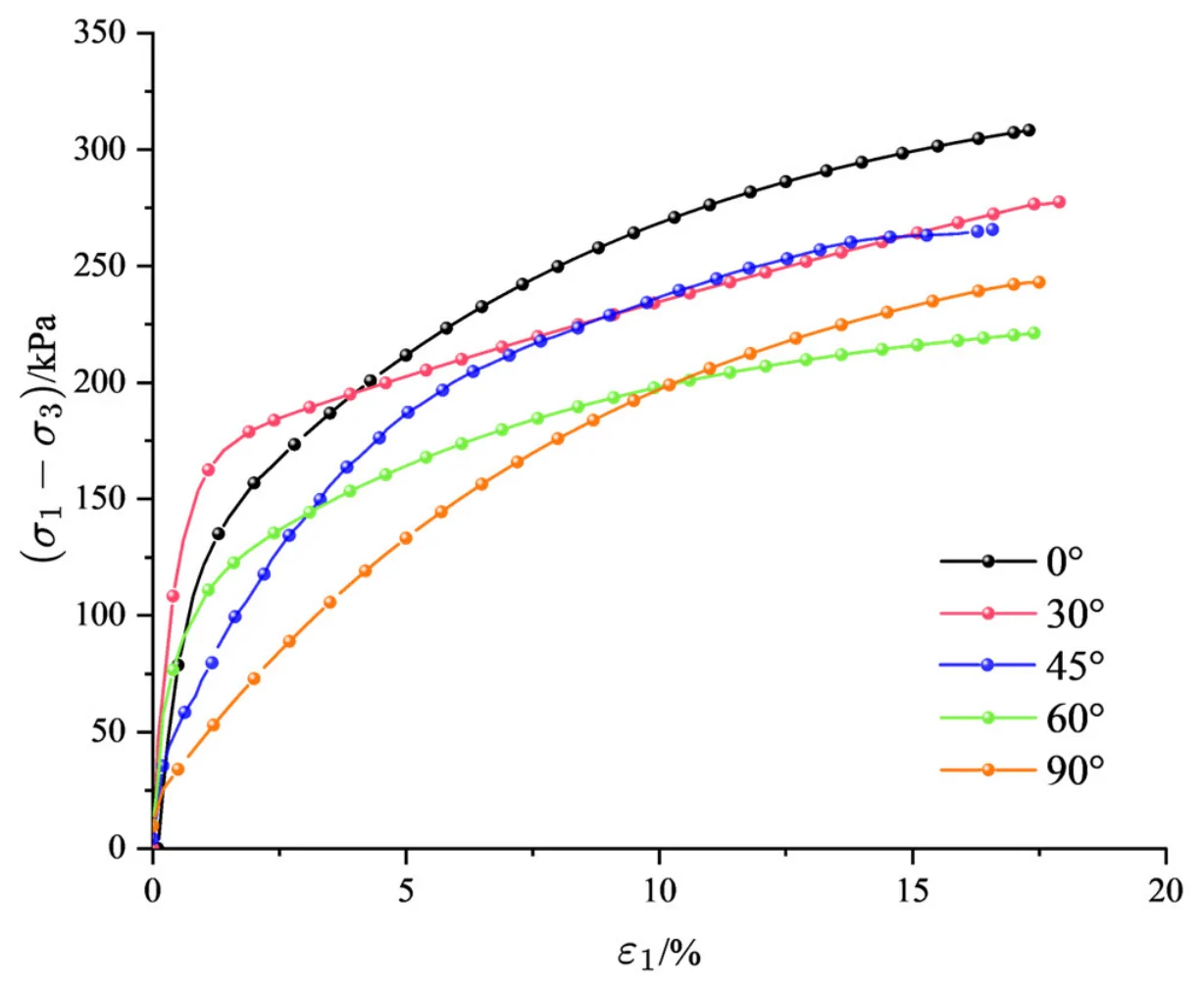
Stress–strain relationship diagram of CCU at consolidation confining pressure of 200 kPa.

As illustrated in Figure 6 and Figure 7, under both consolidation pressures, the stress level adopted as the CCU failure state generally decreases as α increases from 0° to 60° and then increases at α = 90°. The highest adopted failure stress occurs at α = 0°, whereas the lowest occurs at α = 60° at both pressures. The stress–strain curves for different α values are more closely grouped at 100 kPa, whereas greater separation is observed at 200 kPa, indicating that the macroscopic orientation dependence of the tested clay becomes more pronounced at the higher consolidation pressure. A possible interpretation is that increased confinement changes the constraints on particle rearrangement and stress transmission within the soil. However, particle packing, fabric anisotropy, and contact-force distributions were not directly measured in the present study. Therefore, these microstructural mechanisms are considered only as possible explanations for the observed macroscopic pressure–orientation interaction.

The influence of α on deformation should be interpreted as an orientation-dependent response of the specimen relative to the applied principal-stress axes rather than as a transition from compression to tension. Changing α alters the orientation of the deposition-related material direction relative to the applied stress field and may therefore change the resolved normal and shear components acting on material planes. The reported triaxial stress conditions do not provide evidence of a tensile principal-stress state. Therefore, the terms “tensile stress-dominated” and “combined tensile and shear stresses” are not used here. The observed differences in axial-strain development are instead described as macroscopic orientation-dependent responses.

**Figure 8:**
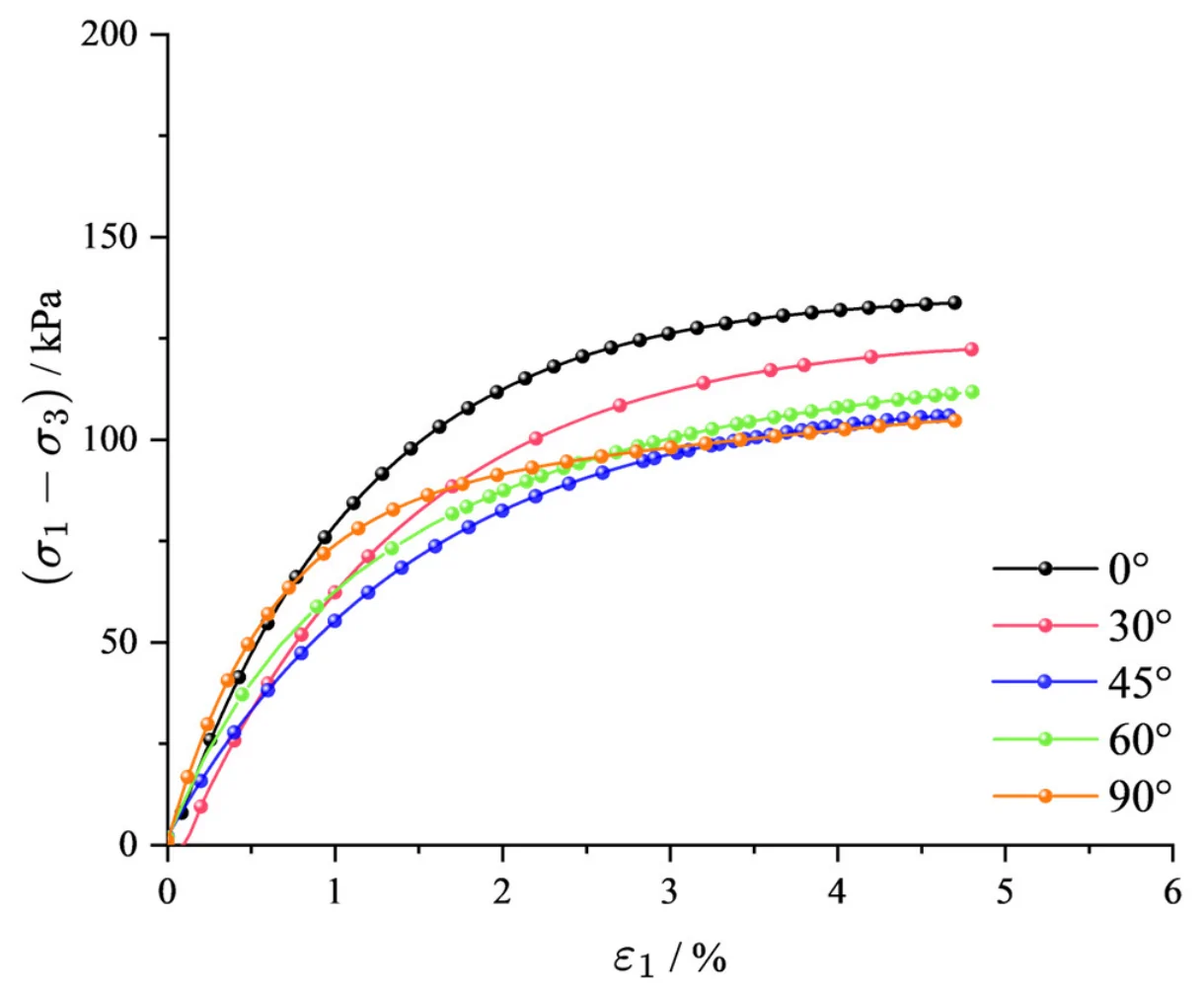
Stress–strain relationship diagram of LUU at consolidation confining pressure of 100 kPa.

**Figure 9:**
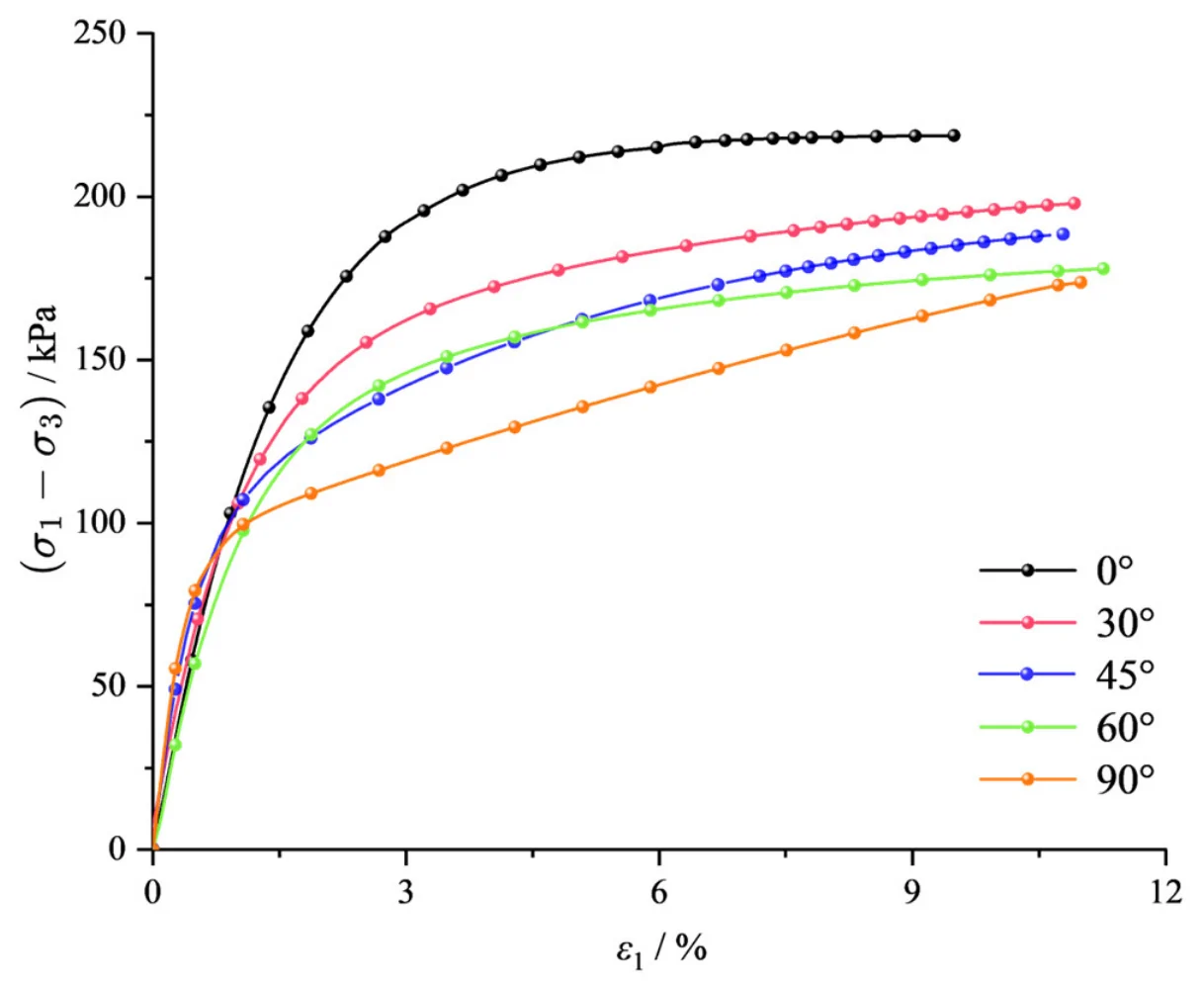
Stress–strain relationship diagram of LUU at consolidation confining pressure of 200 kPa.

As shown in Figure 8 and Figure 9, the measured terminal deviator stress under the LUU path exhibits a pressure-dependent variation with α. At a consolidation pressure of 100 kPa, the terminal deviator stress decreases from α = 0° to 45°, reaches its minimum at α = 45°, increases at α = 60°, and then decreases again at α = 90°, while remaining slightly higher than the value at α = 45°. At 200 kPa, the terminal deviator stress decreases monotonically with increasing α, with the maximum and minimum values occurring at α = 0° and 90°, respectively. Therefore, the two consolidation pressures do not exhibit an identical angular ordering, although α = 0° corresponds to the highest terminal stress in both cases.

Comparison between Figure 8 and Figure 9 further reveals that, under a consolidation confining pressure of 100 kPa, the stress–strain curves corresponding to different α values exhibit relatively small differences and tend to converge. However, when the confining pressure increases to 200 kPa, the curves become increasingly separated, suggesting that higher confinement conditions enhance the dependence of soil mechanical response on the initial principal stress direction. In comparison with the CCU tests, the LUU tests exhibit smaller axial strains at the prescribed unloading endpoints. Because the two protocols have different termination conditions, this difference should not be interpreted as evidence of earlier material failure.

The axial-strain responses under the two test protocols also show different degrees of sensitivity to α. Under the LUU path, the curves at different α values exhibit relatively similar axial-strain development at comparable deviator-stress levels, whereas a clearer orientation dependence is observed in the CCU tests. Accordingly, the present results are interpreted as evidence of different macroscopic orientation sensitivities under the two prescribed stress paths, rather than as direct evidence of specific deformation mechanisms.

### 3.2. Analysis of Shear Strength Relationship

Mohr–Coulomb indices for remolded clay subjected to the CCU and LUU stress paths were derived under different initial principal stress direction angles (α). The Mohr strength envelopes were constructed by taking the shear stress (*τ*) as the ordinate and the normal stress (*σ*) as the abscissa. For each adopted stress state, the radius of the Mohr circle was defined as (*σ*_1_ − *σ*_3_)/2, while the circle center was determined as (*σ*_1_ + *σ*_3_)/2. The operational failure states adopted for the CCU tests and the measured terminal states of the LUU tests were used for the corresponding parameter evaluation. At each orientation angle, only two adopted stress states corresponding to consolidation pressures of 100 and 200 kPa were available. Therefore, the Mohr–Coulomb line was defined directly by these two adopted stress states rather than obtained from a multi-point regression. Because two points uniquely determine a straight line, the goodness of fit and possible nonlinearity of the envelope cannot be evaluated from the present data. Accordingly, the derived c and *φ* values are treated as two-state, apparent Mohr–Coulomb comparison indices over the tested 100–200 kPa range. The corresponding apparent indices are summarized in Table 3.

For consistency with the stress-path representation in Figure 3b, the same Mohr–Coulomb relation can also be expressed in the triaxial p-q plane [26], where $p=\left(\sigma_{1}+2\sigma_{3}\right)/3$ and $q=\left(\sigma_{1}-\sigma_{3}\right)$. The conventional triaxial-compression form of the Mohr–Coulomb criterion can be transformed to:

$$q=\frac{6sin\varphi }{3-sin\varphi }p+\frac{6ccos\varphi }{3-sin\varphi }$$

Thus, the *c* and φ values summarized in Table 3 can be directly used to define the corresponding strength lines in the p-q plane.

The values in Table 3 should therefore be interpreted primarily as comparative indices among orientations and stress paths within the tested 100–200 kPa range.

A comparison of the two-state Mohr–Coulomb indices derived from the CCU and LUU tests (Table 3) shows clear differences between the two test protocols. The apparent cohesion and friction angle derived from the LUU terminal states are generally lower than those obtained from the CCU operational failure states over the tested pressure range. This difference is associated with the distinct stress evolution of the two test protocols: the CCU path maintains lateral confinement during axial shearing, whereas the LUU path involves a progressive reduction in lateral confinement. Accordingly, the differences in the derived indices are interpreted primarily as stress-path-dependent macroscopic responses.

For the CCU stress path, the derived Mohr–Coulomb indices exhibit a systematic dependence on α. The apparent cohesion generally increases with α, whereas the apparent friction angle decreases overall, with a slight increase at α = 90°. These variations are therefore interpreted as macroscopic orientation-dependent trends of the derived indices within the investigated stress range. For the LUU path, the two-state apparent friction angle decreases progressively with increasing α, whereas the apparent cohesion first decreases and then increases, reaching its minimum at α = 45°. These results indicate that the orientation dependence of the derived Mohr–Coulomb indices differs for cohesion and friction angle under lateral unloading.

Overall, the apparent Mohr–Coulomb indices vary with orientation and test protocol over the investigated 100–200 kPa range. The distinct responses observed under the CCU and LUU protocols highlight the importance of incorporating orientation and unloading effects into constitutive descriptions of excavation-related soil behavior.

### 3.3. Analysis of Initial Tangent Modulus and Hyperbolically Fitted Ultimate Deviator Stress

Based on a comprehensive analysis of stress–strain curves obtained from triaxial tests reported in previous studies, Kondner [27] proposed a hyperbolic function to describe the stress–strain behavior of clay under consolidated undrained triaxial loading conditions:

(1) $$\sigma_{1}-\sigma_{3}=\frac{\varepsilon_{1}}{a+b\varepsilon_{1}}$$

where $\sigma_{1}-\sigma_{3}$ is the deviatoric stress, ε_1_ is the axial strain, and *a* and *b* are hyperbolic fitting parameters related to the initial tangent modulus and the fitted ultimate deviator stress, respectively.

Building upon this theoretical framework, Duncan et al. [28,29] further developed the incremental nonlinear elastic constitutive model, commonly known as the Duncan–Chang model. For conventional triaxial compression conditions, the hyperbolic stress–strain relationship can be transformed into a linear form:

(2) $$\frac{\varepsilon_{1}}{\sigma_{1}-\sigma_{3}}=a+b\varepsilon_{1}$$

In the Duncan–Chang model, the initial tangent modulus, defined as *E_i_* = 1/*a*, is used to characterize the initial stiffness of soil under zero strain conditions, while the asymptotic value of the principal stress difference, expressed as (*σ*_1_ *− σ*_3_)*_ult_* = 1/*b*, represents the theoretical ultimate deviator stress approached by the hyperbolic curve. Here, this quantity is a theoretical asymptote obtained by regression and does not represent a directly measured peak, yield, or terminal stress.

**Figure 10:**
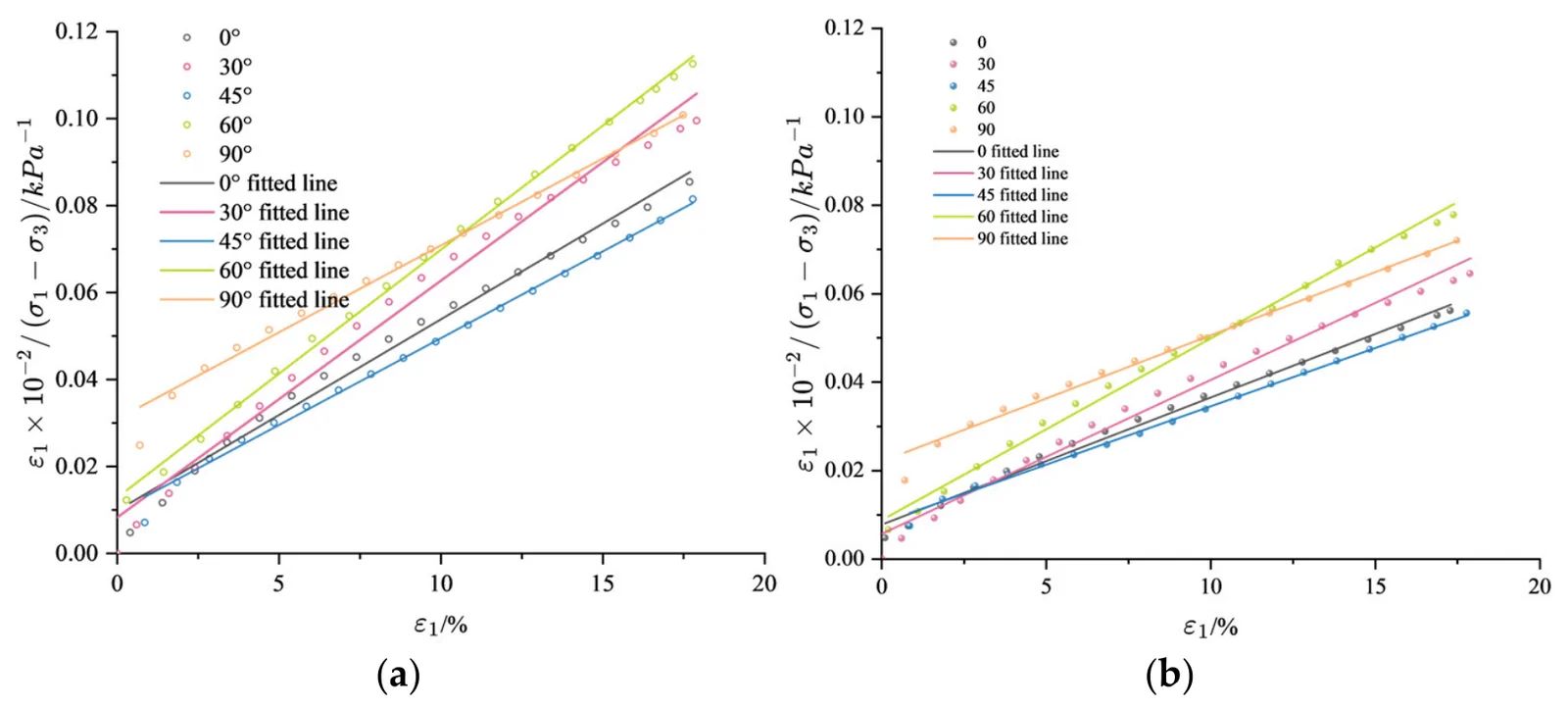
Relationship lines and linear fitting plots of *ε*_1_/(*σ*_1_ − *σ*_3_) versus *ε*_1_ from conventional triaxial compression tests under different initial consolidation confining pressures. (**a**) Consolidation confining pressure of 100 kPa. (**b**) Consolidation confining pressure of 200 kPa.

**Figure 11:**
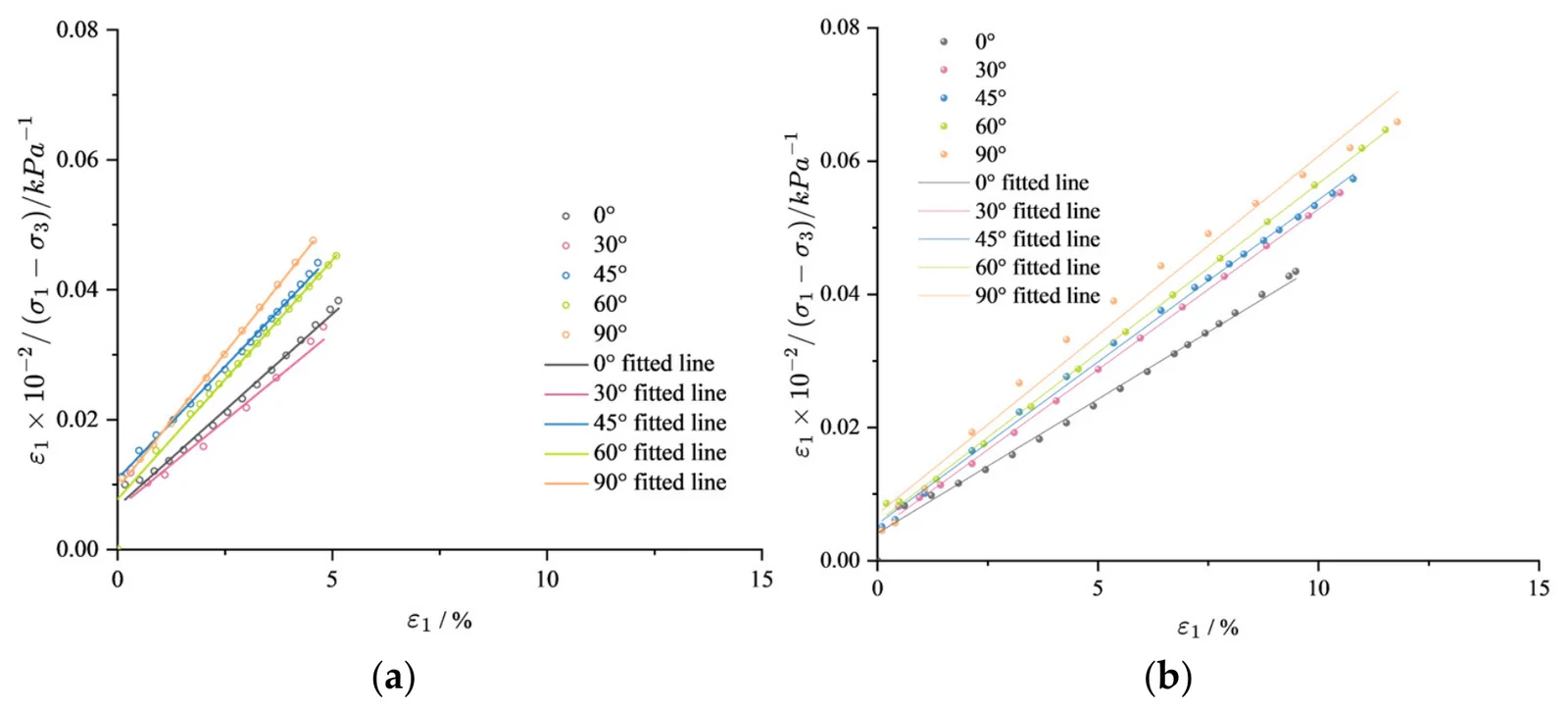
Relationship lines and linear fitting plots of *ε*_1_/(*σ*_1_ − *σ*_3_) versus *ε*_1_ from lateral unloading tests under different initial consolidation confining pressures. (**a**) Consolidation confining pressure of 100 kPa. (**b**) Consolidation confining pressure of 200 kPa.

The linearized $\varepsilon_{1}/\left(\sigma_{1}-\sigma_{3}\right)-\varepsilon_{1}$ relationships obtained from the CCU and LUU tests are presented in Figure 10 and Figure 11. The same linearized fitting procedure was applied to all orientation angles and consolidation pressures under both stress paths. The corresponding adjusted coefficients of determination ($R_{\mathrm{adj}}^{2}$) are summarized in Table 4. The values range from 0.949 to 0.998 for the CCU tests and from 0.908 to 0.999 for the LUU tests, indicating generally good linearity of the transformed stress–strain relationships. These quantitative results support the use of the hyperbolic formulation for describing the measured stress–strain responses under the investigated test conditions.

**Figure 12:**
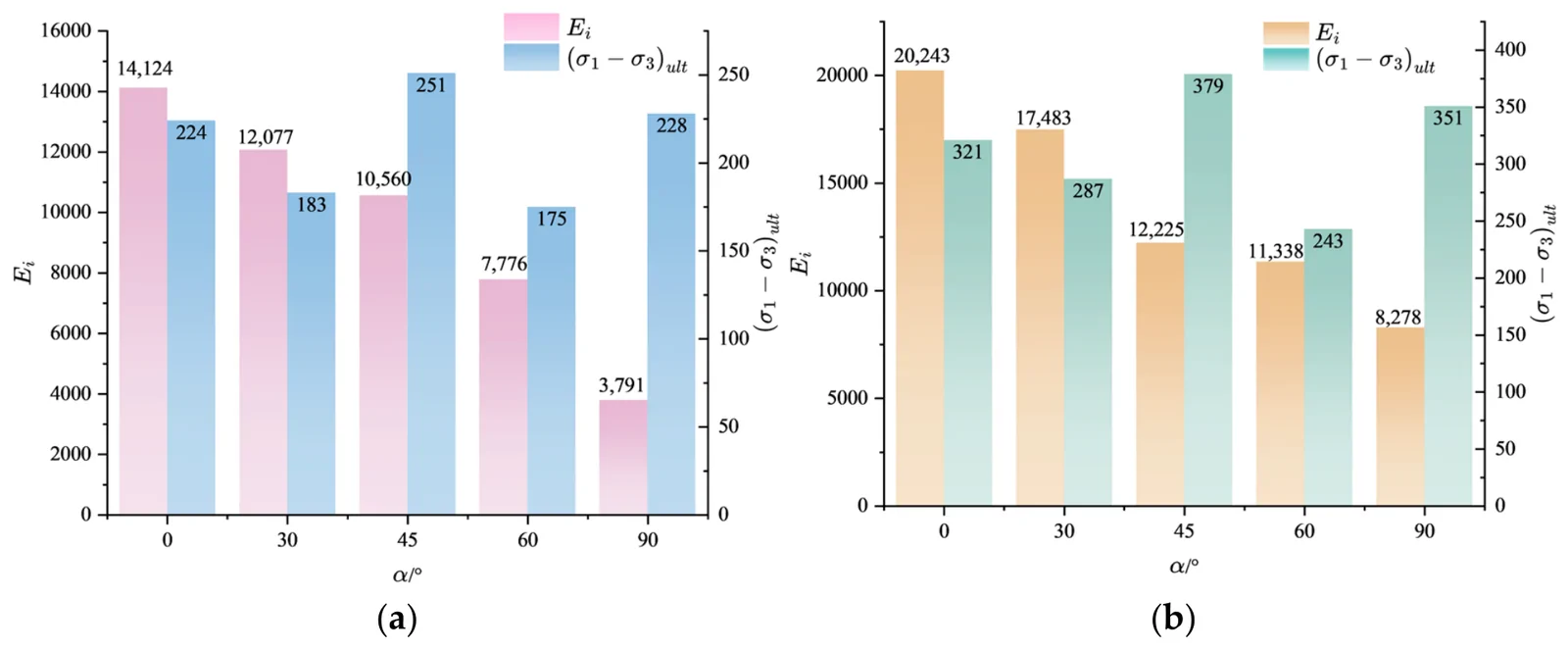
Fitting parameters of the triaxial compression test. (**a**) Consolidation confining pressure of 100 kPa. (**b**) Consolidation confining pressure of 200 kPa.

**Figure 13:**
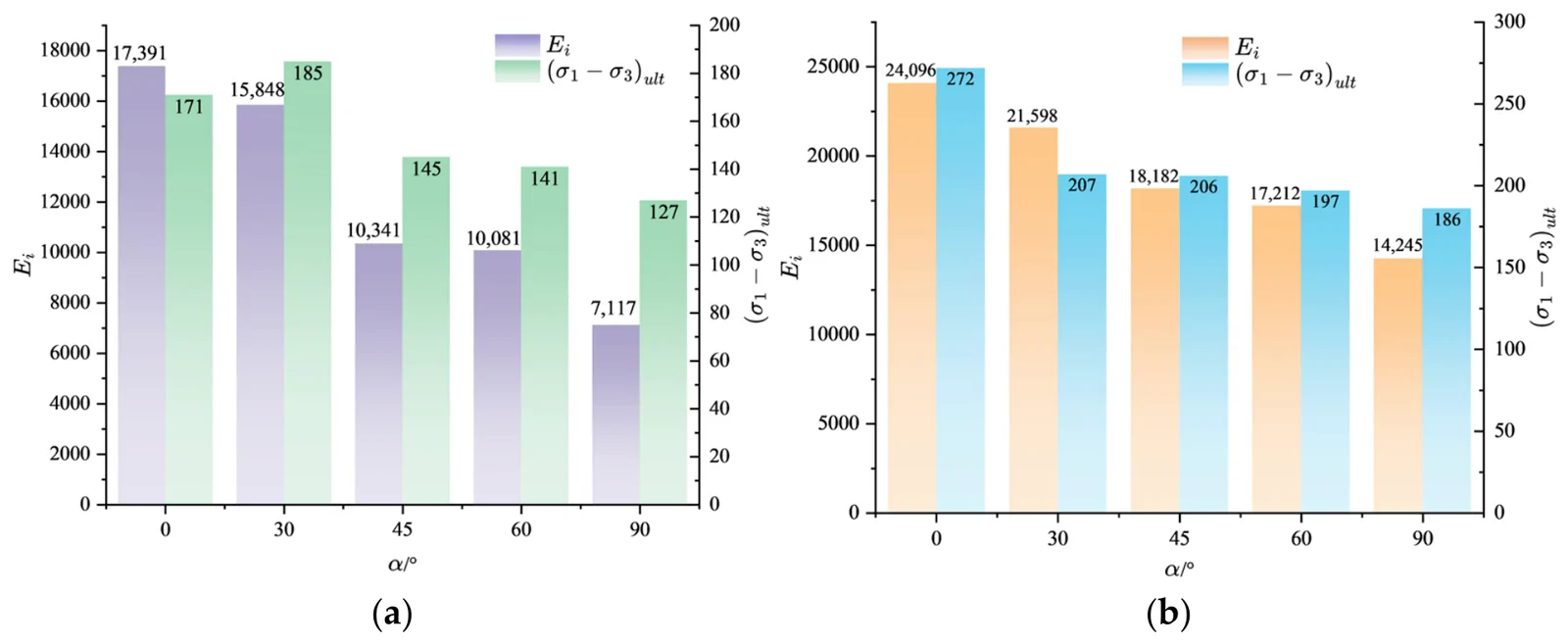
Fitting parameters of lateral unloading test. (**a**) Consolidation confining pressure of 100 kPa. (**b**) Consolidation confining pressure of 200 kPa.

Furthermore, the relationship between *ε*_1_/(*σ*_1_ − *σ*_3_) and *ε*_1_ was linearly fitted to determine the key model parameters, including the initial tangent modulus *E_i_* and the hyperbolically fitted ultimate deviator stress ${\left(\sigma_{1}-\sigma_{3}\right)}_{ult}$, as presented in Figure 12 and Figure 13. The obtained parameters provide essential input for subsequent modification and application of the Duncan–Chang model under unloading stress conditions.

Analysis of the results presented in Figure 10, Figure 11, Figure 12 and Figure 13 demonstrates that increasing the consolidation confining pressure enhances both the initial tangent modulus and the hyperbolically fitted ultimate deviator stress of remolded clay under both the CCU and LUU stress paths. This macroscopic response indicates greater stiffness and a higher fitted stress level at the higher consolidation pressure. The underlying particle-scale mechanisms were not directly investigated in the present study and are therefore not inferred from these results. Under the present testing protocols, the fitted initial tangent modulus obtained from the LUU tests is generally higher than that obtained from the CCU tests at corresponding consolidation pressures and orientation angles. However, the CCU tests were conducted under strain control at 0.05%/min, whereas the LUU tests were conducted under stress control at 0.2 kPa/min. Consequently, the present experimental design does not allow the effects of stress path, control mode, and loading rate on the initial tangent modulus to be independently separated. Therefore, the higher initial tangent modulus observed in the LUU tests should be regarded as a protocol-dependent experimental observation rather than direct evidence of an intrinsic stiffening effect caused solely by lateral unloading. In contrast, comparisons among different α values within the same stress path were conducted using the same control mode and rate and are therefore more appropriate for evaluating the effect of orientation. However, despite the higher initial stiffness, the hyperbolically fitted ultimate deviator stress for the LUU tests is generally lower than that for the CCU tests, indicating a lower fitted asymptotic stress level under the LUU protocol.

A comparison between the CCU and LUU test results further reveals distinct effects of the initial principal stress direction angle α on the initial tangent modulus and hyperbolically fitted ultimate deviator stress under the same consolidation confining pressure. For the CCU stress path, the initial tangent modulus exhibits a monotonic decreasing trend with increasing α, reaching its maximum value at α = 0°. This indicates that the soil exhibits the greatest initial stiffness when the major principal stress direction coincides with the soil deposition direction. In contrast, the hyperbolically fitted ultimate deviator stress presents a non-monotonic variation with α, characterized by an initial decrease, followed by an increase, and subsequently a reduction, with the maximum value occurring at α = 45°. These results show that the initial stiffness and the fitted asymptotic stress exhibit different orientation-dependent trends under the CCU protocol. In particular, the maximum fitted ultimate deviator stress at α = 45° is reported here as an experimentally observed orientation-dependent feature.

Under the LUU path, the fitted ultimate deviator stress shows an overall decreasing tendency with increasing α, although the variation is not strictly monotonic. At 100 kPa, the fitted ultimate deviator stress increases from 171 kPa at α = 0° to 185 kPa at α = 30° and subsequently decreases to 127 kPa at α = 90°. At 200 kPa, it decreases monotonically from 272 kPa at α = 0° to 186 kPa at α = 90°. These results demonstrate a clear orientation dependence of the fitted LUU parameters within the investigated range. The measured LUU response is therefore sensitive to the initial orientation relative to the principal-stress axes, supporting the introduction of orientation-dependent parameters in the subsequent constitutive formulation.

## 4. Orientation-Dependent LUU Duncan–Chang Extension Considering the Initial Major Principal Stress Direction

The Duncan–Chang model describes the nonlinear stress–strain behavior of soils based on the incremental generalized Hooke’s law, with its fundamental formulation derived from fitting conventional triaxial compression stress–strain curves using the hyperbolic relationship proposed by Kondner [27]. Its advantages include a relatively small number of parameters, clear physical meaning, and convenient implementation in engineering analyses. However, the conventional Duncan–Chang formulation does not explicitly consider stress path dependency, loading–unloading criteria, or directional effects, which may limit its applicability under complex stress conditions.

In this study, the Duncan–Chang framework is adopted as the basis for extension because the *ε*_1_/(*σ*_1_ − *σ*_3_) ~ *ε*_1_ relationships obtained from the lateral unloading tests maintain an approximately linear trend, indicating that the hyperbolic assumption remains applicable under the investigated unloading condition. Duncan et al. [29] further extended the Duncan–Chang framework for nonlinear soil analysis and finite-element applications by incorporating stress-dependent stiffness and strength parameters. Relative to this broader stress-dependent formulation, the present extension has a different specific objective: it specializes the hyperbolic tangent-modulus relation to the prescribed LUU path and treats fixed material orientation as an explicit conditioning variable in the parameterization. Building upon this framework, the present study develops an orientation-dependent LUU Duncan–Chang extension for the prescribed lateral unloading undrained (LUU) stress path. The proposed extension retains the incremental stress–strain formulation while introducing orientation-dependent parameters *K*(α), *n*(α), *R_f_*(α), *c*(α), and $\varphi \left(\alpha \right)$ to represent the influence of the initial major principal stress direction on unloading deformation behavior.

### 4.1. Orientation-Dependent LUU Duncan–Chang Extension

For the prescribed axisymmetric LUU path, the incremental generalized Hooke-type relation is retained only as a local algebraic reference at each fixed orientation α; it is not interpreted as an orientation-independent description of the clay. Compression is taken as positive. During LUU, *σ*_1_ is the axial total principal stress, $\sigma_{2}=\sigma_{3}$ are the radial total principal stresses, $q=\sigma_{1}-\sigma_{3}$ is the deviator stress, and $\varepsilon_{1}$ is the measured axial strain. The axial total principal stress at the onset of lateral unloading is denoted by *σ*_1,0_, and *σ*_3,*ref*_ denotes the radial total principal stress at the reference state used to define the reference deviator stress *q_ref_*. The effective isotropic consolidation pressure at the end of drained consolidation is denoted by $p_{c}^{\prime }$ and is used only in the initial-modulus scaling. Accordingly, *σ*_1_, *σ*_3_, *σ*_1,0_, *σ*_3,*ref*_, and *q* in the LUU stress-level normalization are expressed in the same total-stress reference used in Section 3.2, whereas $p_{c}^{\prime }$ is an effective-stress quantity used only to represent the consolidation level in the initial-modulus relation. $E_{H}$ denotes the working tangent modulus in the local Hooke-type relation, $\nu_{t}$ is the corresponding tangent Poisson ratio, $E_{u,t}=dq/d\varepsilon_{1}$ is the experimentally defined LUU path tangent modulus, and $E_{i}$ is the initial tangent modulus obtained from the hyperbolic fitting in Section 3.3.

For the local incremental reference relation, the three normal strain increments are:

(3) $$d\varepsilon_{1}=\frac{1}{E_{H}}\left[d\sigma_{1}-\nu_{t}\left(d\sigma_{2}+d\sigma_{3}\right)\right]$$

(4) $$d\varepsilon_{2}=\frac{1}{E_{H}}\left[d\sigma_{2}-\nu_{t}\left(d\sigma_{1}+d\sigma_{3}\right)\right]$$

(5) $$d\varepsilon_{3}=\frac{1}{E_{H}}\left[d\sigma_{3}-\nu_{t}\left(d\sigma_{1}+d\sigma_{2}\right)\right]$$

For the prescribed axisymmetric LUU increment, the axial stress is maintained approximately constant while the two radial stresses decrease equally:

(6) $$d\sigma_{1}\approx 0,\;\;d\sigma_{2}=d\sigma_{3}=d\sigma_{r}<0$$

Because $q=\sigma_{1}-\sigma_{3}$, the corresponding deviator-stress increment is:

(7) $$dq=d\left(\sigma_{1}-\sigma_{3}\right)=d\sigma_{1}-d\sigma_{3}\approx -d\sigma_{r}>0$$

The LUU path tangent modulus measured from the $q-\varepsilon_{1}$ response is therefore defined as:

(8) $$E_{u,t}\equiv \frac{dq}{d\varepsilon_{1}}\approx -\frac{d\sigma_{r}}{d\varepsilon_{1}}$$

Substituting the LUU stress increments into the local Hooke-type relation gives the axial and radial strain increments:

(9) $$d\varepsilon_{1}=-\frac{2\nu_{t}}{E_{H}}d\sigma_{r}$$

(10) $$d\varepsilon_{r}=d\varepsilon_{2}=d\varepsilon_{3}=\frac{1-\nu_{t}}{E_{H}}d\sigma_{r}$$

The associated volumetric strain increment is:

(11) $$d\varepsilon_{v}=d\varepsilon_{1}+2d\varepsilon_{r}=\frac{2\left(1-2\nu_{t}\right)}{E_{H}}d\sigma_{r}$$

For the saturated undrained shear stage, the specimen volume is taken as approximately constant within this local algebraic closure:

(12) $$d\varepsilon_{v}\approx 0$$

(13) $$\nu_{t}\approx 0.5$$

Combining the path-tangent definition with the axial strain increment gives:

(14) $$E_{u,t}=\frac{E_{H}}{2\nu_{t}}$$

(15) $$E_{H}=2\nu_{t}E_{u,t}\approx E_{u,t}$$

Thus, for the saturated undrained LUU increment, the working modulus *E_H_* in the local Hooke-type reference and the experimentally observed path tangent $E_{u,t}$ are approximately equivalent. The approximation $\nu_{t}\approx 0.5$ is used only as the zero-volume-change closure of the local incremental relation and is not introduced as an independently measured Poisson ratio.

The measured LUU stress–strain response is then represented by the Kondner hyperbola:

(16) $$q=\frac{\varepsilon_{1}}{a+b\varepsilon_{1}}$$

(17) $$\frac{\varepsilon_{1}}{q}=a+b\varepsilon_{1}$$

Differentiation with respect to axial strain defines the LUU tangent modulus and gives:

(18) $$E_{u,t}=\frac{dq}{d\varepsilon_{1}}$$

(19) $$E_{u,t}=\frac{a}{{\left(a+b\varepsilon_{1}\right)}^{2}}$$

The two hyperbolic constants define the initial tangent modulus and the fitted asymptotic deviator stress:

(20) $$E_{i}=\frac{1}{a}$$

(21) $$q_{ult}=\frac{1}{b}$$

Here, *a* is the intercept, and *b* is the slope of the linearized $\varepsilon_{1}/q$ − $\varepsilon_{1}$ relation. Accordingly, $q_{ult}=1/b$ is a hyperbolically fitted asymptotic stress rather than a directly measured peak, yield, or terminal stress. Using these definitions, the path tangent modulus becomes:

(22) $$E_{u,t}=E_{i}{\left(1-\frac{q}{q_{ult}}\right)}^{2}$$

Following the pressure-dependent initial-modulus form adopted in the Duncan–Chang framework [28,30], the initial tangent modulus is expressed as:

(23) $$E_{i}=Kp_{a}{\left(\frac{p_{c}^{\prime }}{p_{a}}\right)}^{n}$$

where *p*_a_ is the reference pressure (100 kPa in the present parameter identification), $p_{c}^{\prime }$ is the effective isotropic consolidation pressure, *K* is the dimensionless modulus number, and *n* is the pressure exponent. The logarithmic form is:

(24) $$lg\left(\frac{E_{i}}{p_{a}}\right)=lgK+nlg\left(\frac{p_{c}^{\prime }}{p_{a}}\right)$$

Thus, *n* is the slope of the $lg\left(E_{i}/p_{a}\right)-lg\left(p_{c}^{\prime }/p_{a}\right)$ relation, whereas lgK is its intercept. This definition is used consistently for the parameter determination in Section 4.2.

To introduce a strength-normalized stress level without identifying the measured LUU endpoint with a peak failure state, the two-state apparent Mohr–Coulomb indices *c* and *φ* from Section 3.2 are used to define a reference deviator stress $q_{ref}$. The corresponding LUU stress level is:

(25) $$S_{LUU}\equiv \frac{q}{q_{ref}}$$

The reference stress $q_{ref}$ is a model-normalization quantity; it is distinct from both the measured terminal deviator stress and the hyperbolic asymptote $q_{ult}$. In the same stress reference used for the apparent Mohr–Coulomb analysis, the reference state satisfies:

(26) $$\frac{q_{ref}}{2}=ccos\varphi +\frac{\sigma_{1,0}+\sigma_{3,ref}}{2}sin\varphi$$

Because the axial principal stress remains approximately constant during the prescribed LUU path, the radial reference stress can be written as:

(27) $$\sigma_{3,ref}=\sigma_{1,0}-q_{ref}$$

Substituting Equation (27) into Equation (26) gives:

(28) $$q_{ref}=\frac{2\sigma_{1,0}sin\varphi +2ccos\varphi }{1+sin\varphi }$$

Hence, the apparent strength-normalized LUU stress level becomes:

(29) $$S_{LUU}=\frac{q\left(1+sin\varphi \right)}{2\sigma_{1,0}sin\varphi +2ccos\varphi }$$

The exact hyperbolic tangent relation in Equation (22) is governed by $q/q_{ult}$. To retain the Duncan–Chang stress-level structure while using the LUU strength normalization, an orientation-specific dimensionless mapping factor *R_f_* is introduced:

(30) $$\eta \equiv \frac{q}{q_{ult}}\approx R_{f}S_{LUU}$$

The numerical *R_f_* values used in the present formulation were retained from the original Duncan–Chang-based parameter reduction of the LUU tests. In that reduction, *R_f_* followed the conventional Duncan–Chang failure-ratio definition, in which an adopted reference/failure deviator stress is related to the hyperbolically fitted asymptotic deviator stress *q_ult_*. The *q_ult_* values were obtained from the Kondner fits to the measured LUU deviator-stress–axial-strain responses, and one orientation-specific *R_f_* value was reported for the 100 and 200 kPa test cases at each fixed orientation. In the present formulation, these previously determined numerical values are retained, while Equation (30) uses *R_f_* as the coefficient relating the LUU-specific normalized stress level *S_LUU_* to the hyperbolic stress ratio *q*/*q_ult_*.

The symbol *R_f_* is retained for continuity with the conventional Duncan–Chang framework. In that framework, *R_f_* is introduced as a failure-ratio parameter relating an adopted failure/reference deviator stress to the hyperbolic asymptotic deviator stress. Because the present LUU curves are strain-hardening and do not exhibit a distinct peak-failure state, the retained *R_f_* values are not re-estimated from, or interpreted as ratios involving, the measured LUU terminal stresses. Instead, they are used here only as orientation-specific empirical coefficients that map the apparent LUU strength-normalized stress level *S_LUU_* to the hyperbolic stress ratio $q/q_{ult}$ in Equation (30).

With this mapping, Equation (22) becomes:

(31) $$E_{u,t}\approx E_{i}{\left(1-R_{f}S_{LUU}\right)}^{2}$$

Introducing the pressure-dependent initial-modulus relation from Equation (23) gives:

(32) $$E_{u,t}\approx Kp_{a}{\left(\frac{p_{c}^{\prime }}{p_{a}}\right)}^{n}{\left(1-R_{f}S_{LUU}\right)}^{2}$$

Finally, substituting the explicit LUU stress level from Equation (29) and writing the orientation-dependent parameters as functions of α gives the working tangent-modulus relation:

(33) $$E_{u,t}\left(\alpha \right)\approx K\left(\alpha \right)p_{a}{\left(\frac{p_{c}^{\prime }}{p_{a}}\right)}^{n\left(\alpha \right)}{\left[1-R_{f}\left(\alpha \right)\frac{q\left[1+sin\varphi \left(\alpha \right)\right]}{2\sigma_{1,0}sin\varphi \left(\alpha \right)+2c\left(\alpha \right)cos\varphi \left(\alpha \right)}\right]}^{2}$$

Equation (33) is the orientation-dependent tangent-modulus expression adopted for the prescribed saturated LUU path. The term $Kp_{a}{\left(\frac{p_{c}^{\prime }}{p_{a}}\right)}^{n}$ represents the initial stiffness associated with the consolidation state, whereas ${\left(1-R_{f}S_{LUU}\right)}^{2}$ represents the stress-dependent reduction of tangent stiffness during lateral unloading. The orientation effect is incorporated through *K*(α), *n*(α), *R_f_*(α), *c*(α), and φ(α). For each tested orientation, the corresponding parameter values are first derived from the experimental LUU responses or their associated parameter reduction procedures. These discrete orientation-specific values are subsequently represented by empirical functions of α in Section 4.2 to construct the continuous orientation-dependent formulation.

For clarity, the fixed-orientation parameters used in the present formulation have different origins. The hyperbolic parameters *q_ult_* and *E_i_* are obtained from the Kondner fits of the measured LUU stress–strain responses, while *c* and φ are derived as two-state apparent Mohr–Coulomb indices from the experimental stress states. The parameter *R_f_* is retained from the Duncan–Chang-based reduction in the experimental responses, as clarified above. These fixed-orientation quantities constitute the identification basis of the proposed LUU formulation, whereas the subsequent orientation-dependent functions represent empirical constitutive parameterizations.

### 4.2. Analysis of Parameters for the Orientation-Dependent LUU Duncan–Chang Extension

**Figure 14:**
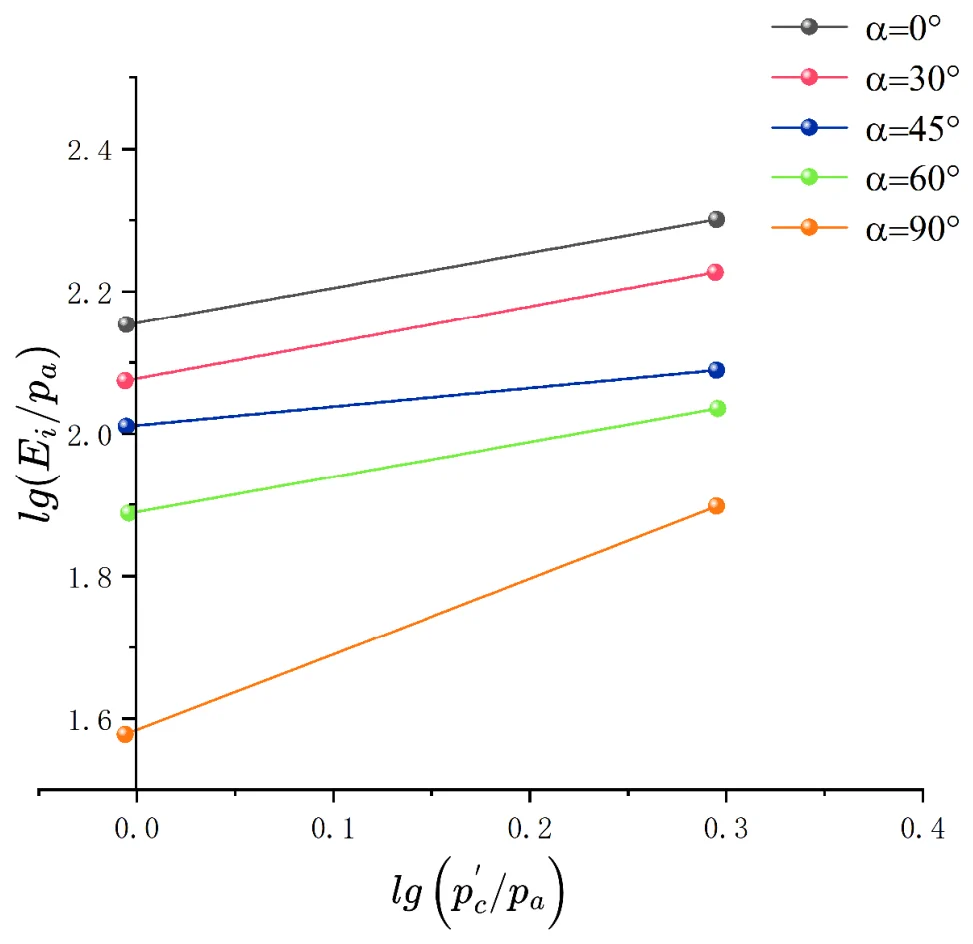
Logarithmic two-pressure relation used to identify K and *n* for the five fixed orientations.

Using the initial tangent modulus *Ei* obtained from the LUU hyperbolic fits, *K* and *n* were determined at each orientation from the $lg\left(E_{i}/p_{a}\right)$ − $lg\left(p_{c}^{\prime }/p_{a}\right)$ relation in Equation (24), with $p_{a}=$ 100 kPa and $p_{c}^{\prime }=$ 100 or 200 kPa. Accordingly, $K=E_{i}\left(p_{c}^{\prime }=100\;\mathrm{kPa}\right)/p_{a}$, and *n* is the two-pressure logarithmic slope. The resulting relations are illustrated in Figure 14.

**Figure 15:**
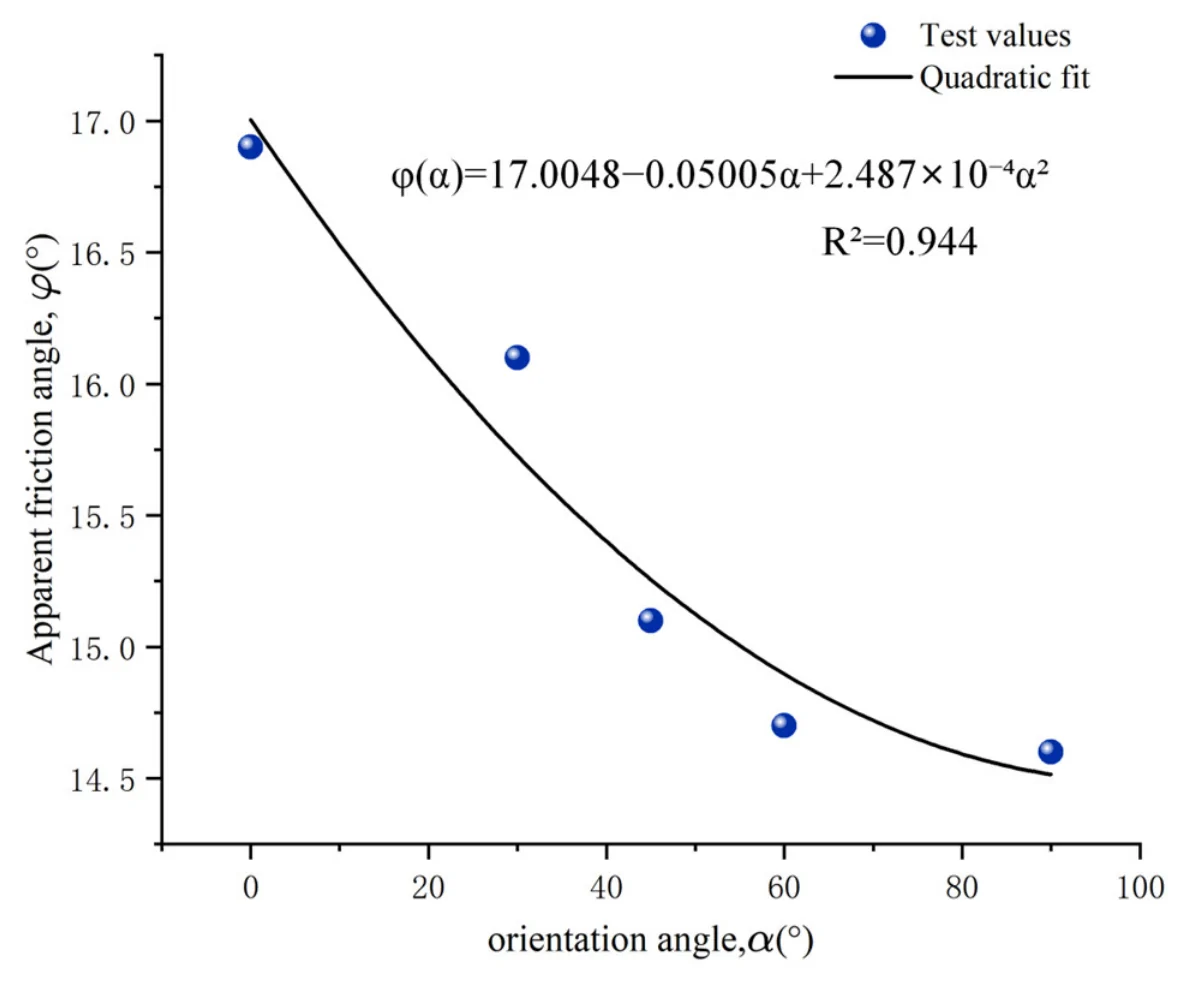
Variations in the apparent LUU friction angle with orientation angle α.

**Figure 16:**
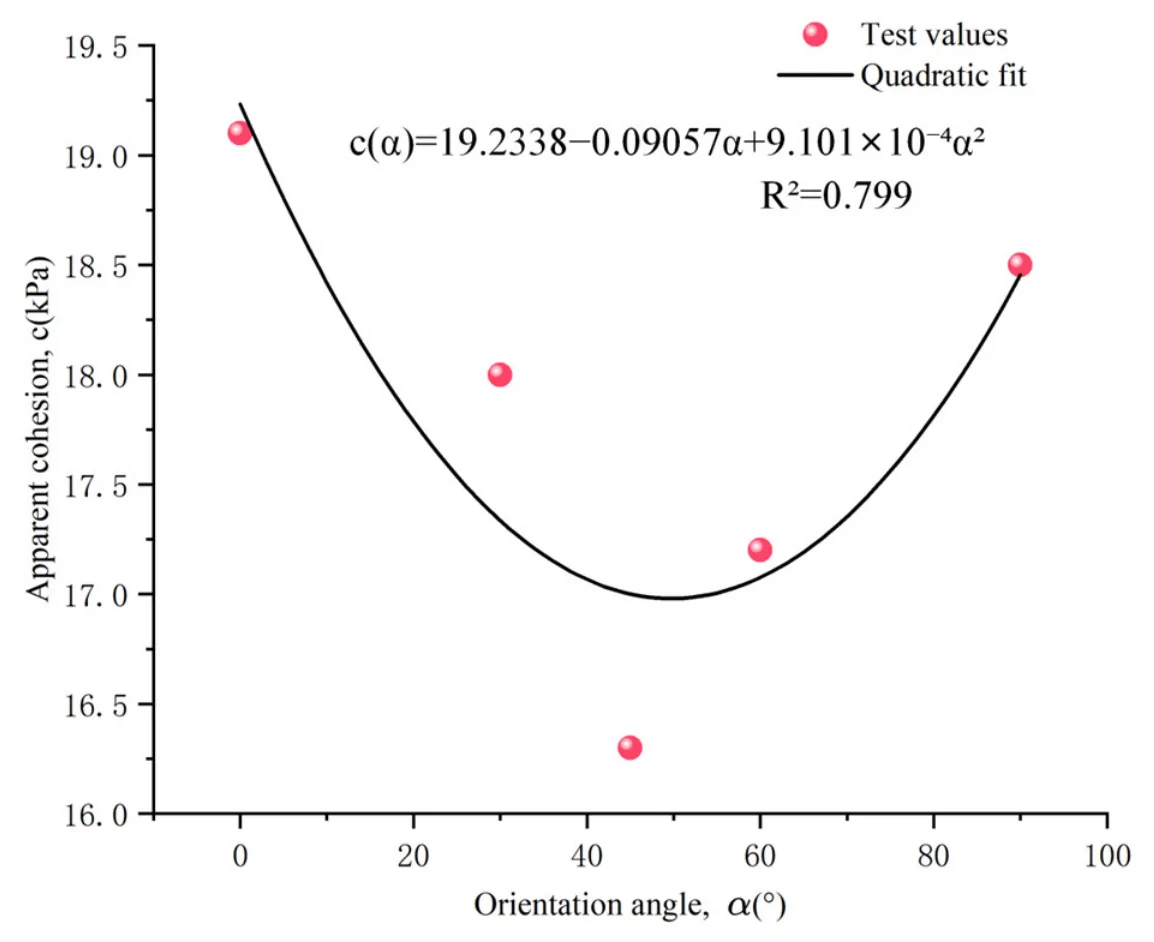
Variations in the apparent LUU cohesion with orientation angle α.

**Figure 17:**
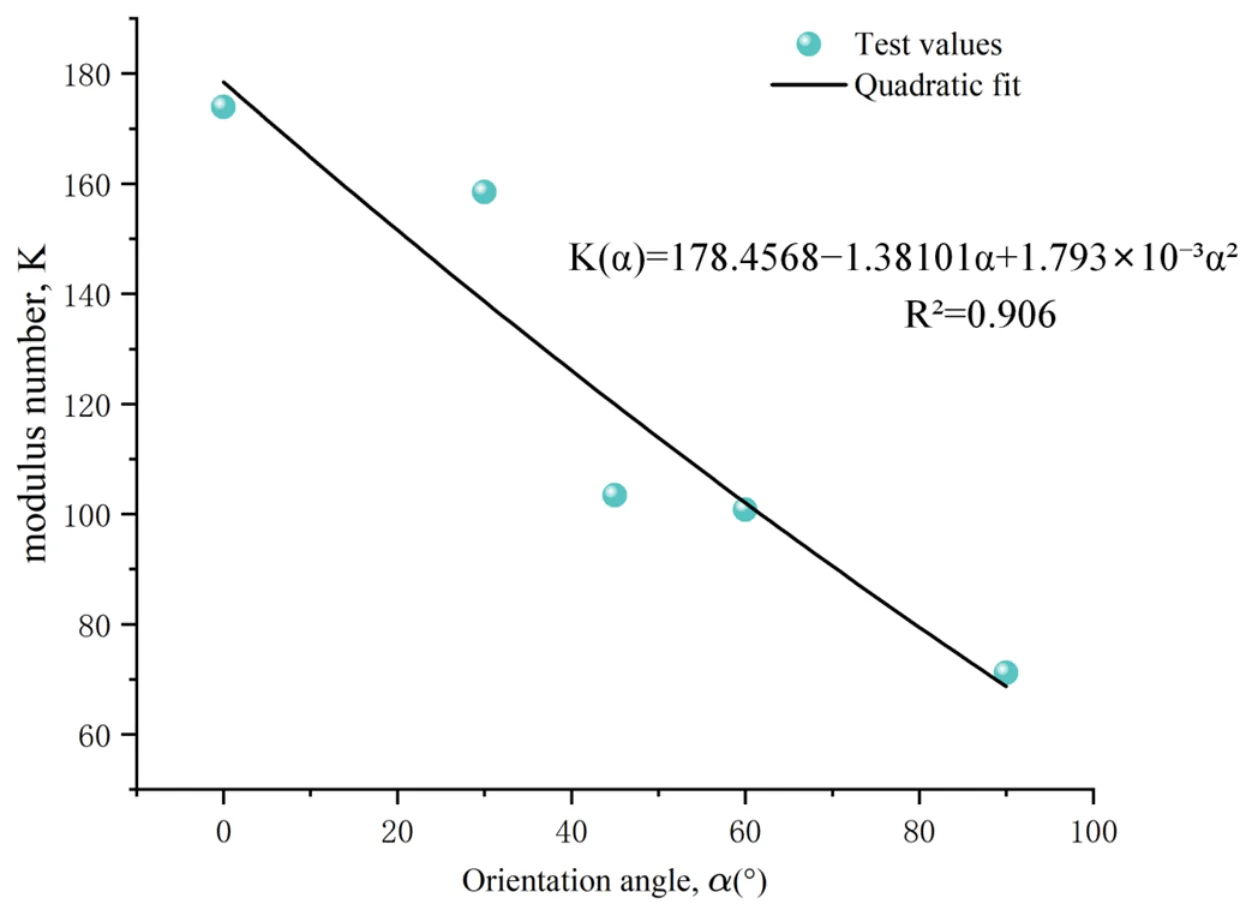
Relationship between modulus number *K* and orientation angle α.

**Figure 18:**
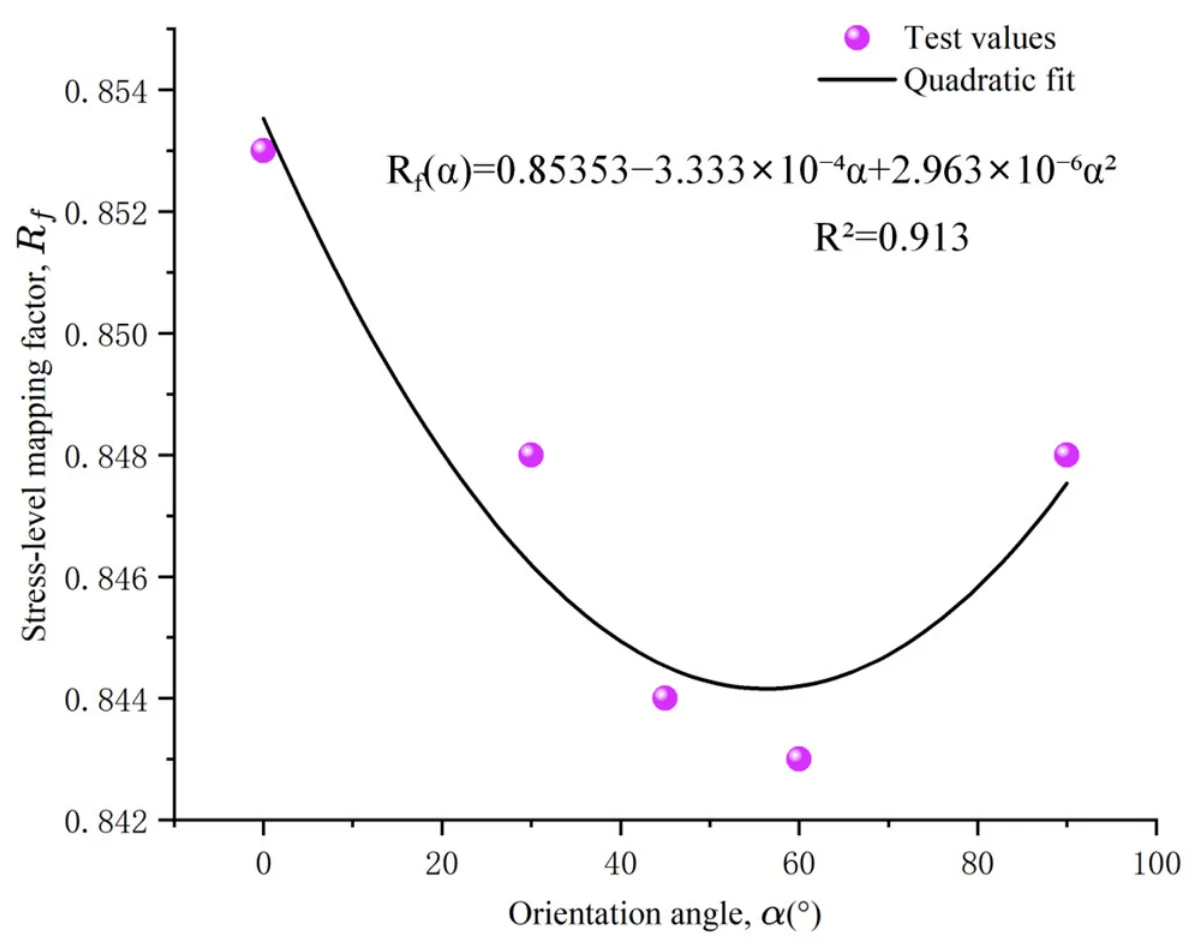
Variations in the stress-level mapping factor *R_f_* with orientation angle α.

**Figure 19:**
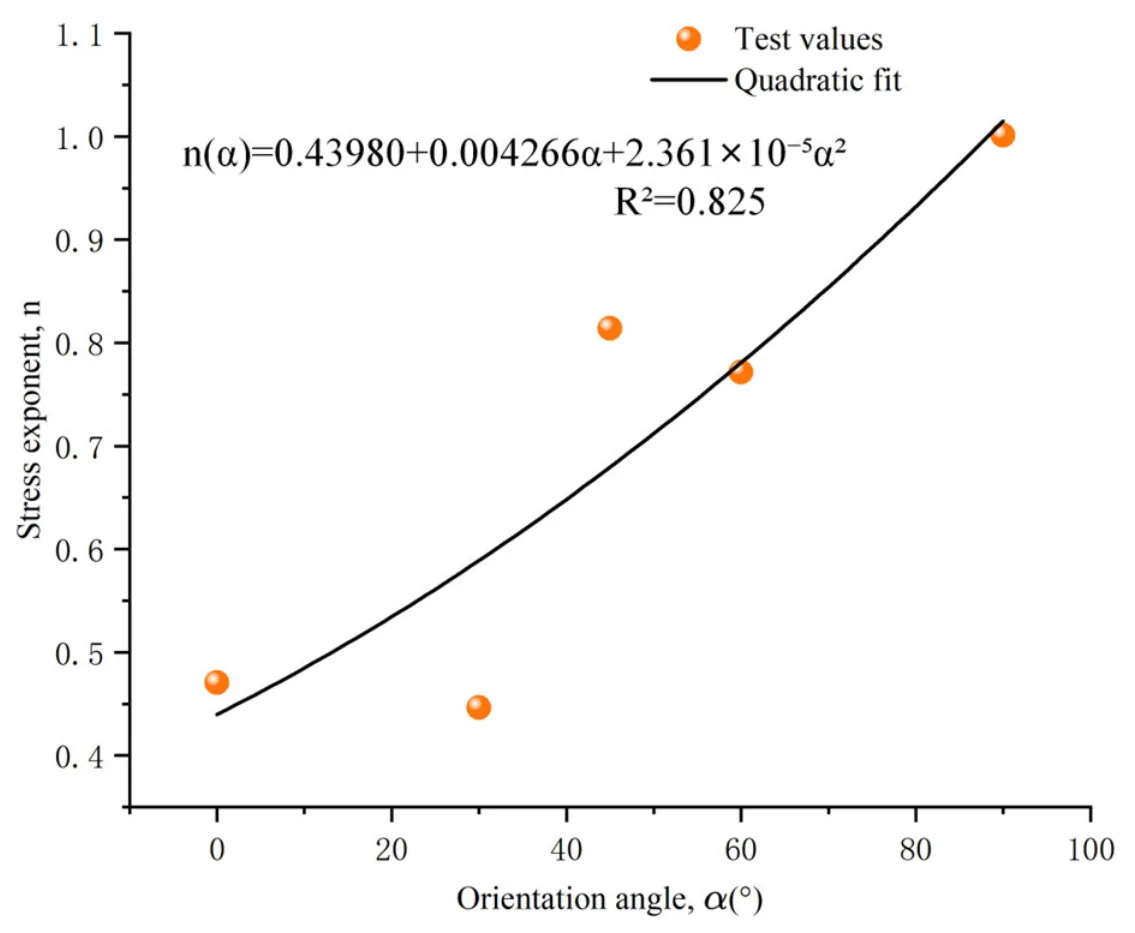
Relationship between stress exponent *n* and orientation angle α.

The identified fixed-orientation quantities summarized in Table 5 were subsequently used to construct the orientation-dependent formulation. The relationships *K*(α), *n*(α), *R_f_*(α), *c*(α), and φ(α) were represented using second-order polynomial interpolation over the investigated 0–90° range, as shown in Figure 15, Figure 16, Figure 17, Figure 18 and Figure 19.

The resulting orientation-specific coefficients are *R_f_* = 0.853, 0.848, 0.844, 0.843, and 0.848 for α = 0°, 30°, 45°, 60°, and 90°, respectively. Their use in the present LUU formulation is consistent with two features of the experimental results. First, the LUU responses retain the hyperbolic characteristics underlying the Duncan–Chang framework, as indicated by the generally good linearity of the Kondner transformations ($R_{adj}^{2}$ = 0.908–0.999). Second, the internal orientation hold-out check predicted *R_f_* = 0.8449 at the withheld α = 60°, compared with the reference value of 0.8430, corresponding to an absolute relative deviation of 0.2%. The latter result supports interpolation of *R_f_*(α) within the investigated angular range. Accordingly, *R_f_*(α) is used here as an empirical orientation-conditioned stress-level mapping coefficient for the tested saturated LUU path and the investigated 100–200 kPa consolidation-pressure range.

The parameters in Table 5 have different identification routes. The parameters *q_ult_* and *E_i_* were obtained from Kondner fitting of the measured LUU stress–strain responses; *c* and φ were derived as two-state apparent Mohr–Coulomb indices from the experimental stress states; *K* and *n* were calculated from the pressure-dependent initial modulus relationship using the fitted *E_i_* values; and *R_f_* was retained as the Duncan–Chang stress-level mapping coefficient described in Section 4.1. The orientation-dependent functions were obtained by empirical interpolation of these fixed-orientation values rather than direct measurements.

The orientation-dependent parameters exhibit distinct empirical trends over α = 0–90°. With the normalization in Equation (23), the modulus number *K* decreases from 173.91 at α = 0° to 71.17 at α = 90°, consistent with the overall reduction in the fitted initial tangent modulus. Figure 17 summarizes this variation.

The pressure exponent *n* quantifies the sensitivity of *E_i_* to the consolidation-pressure change from 100 to 200 kPa at each fixed orientation. The corrected values are 0.470, 0.447, 0.814, 0.772, and 1.001 for α = 0°, 30°, 45°, 60°, and 90°, respectively. Thus, *n* shows an overall increase with α while retaining local non-monotonic variations between adjacent orientations; Figure 19 presents the corresponding empirical trend.

The stress-level mapping factor *R_f_* varies within a narrow range (0.843–0.853) and reaches its minimum near α = 45–60°. The conventional symbol *R_f_* is retained for continuity with the Duncan–Chang framework, while its role in the present formulation is the orientation-specific mapping introduced in Equation (30). Figure 18 shows its variation with α.

Overall, the fixed-orientation quantities provide the identification basis for the proposed LUU formulation, whereas the fitted α-dependent functions provide the constitutive representation of their orientation variation. Together with Equation (33), these empirical relationships enable continuous parameter estimation within the investigated orientation range of 0–90°, while preserving the distinction between quantities identified from the test data and the model parameterizations constructed from them.

### 4.3. Internal Orientation Hold-Out Check

Overall, the orientation-dependent parameters were first established from the tested orientations and then assessed by an internal hold-out interpolation check. In this check, the boundary orientations α = 0° and 90° were retained, while the intermediate orientation α = 60° was withheld from the calibration set. The *c*, *φ*, *K*, *n*, and *R_f_* relationships were refitted using only α = 0°, 30°, 45°, and 90°, and the resulting functions were then used to predict the parameter set at α = 60°. This procedure evaluates interpolation within the investigated angular domain while retaining the same material, consolidation-pressure range, and LUU protocol.

The predicted and reference values for the held-out α = 60° condition are summarized in Table 6. The absolute relative deviations were 1.1% for *c*, 2.1% for *φ*, 1.9% for *K*, 1.8% for *n*, and 0.2% for *R_f_*. The predicted initial tangent moduli derived from the held-out *K* and *n* values were 10,274 kPa at 100 kPa consolidation pressure and 17,714 kPa at 200 kPa, compared with 10,081 and 17,212 kPa, respectively, obtained from the α = 60° test; the corresponding deviations were 1.9% and 2.9%.

These results support the interpolation capability of the orientation-conditioned parameterization at an uncalibrated intermediate orientation within the present test matrix. The particularly small deviation in *R_f_* should be interpreted cautiously because *R_f_* varies only within a narrow numerical range. The hold-out exercise is an internal validation of the parameter-angle relationships rather than an independent validation of the complete stress–strain response or field-scale model performance. After the hold-out check, all five measured orientations were used for the final parameterization shown in Figure 15, Figure 16, Figure 17, Figure 18 and Figure 19.

## 5. Discussion

A key implication of the present results is that the mechanical response under a prescribed lateral unloading path remains dependent on the fixed orientation of the specimen relative to the deposition direction. Previous studies have established that excavation-induced stress relief and unloading history can substantially alter soil stress paths, stiffness, and deformation responses [7,17,18,19], while other investigations have demonstrated the importance of principal-stress direction in the mechanical response of geomaterials [8,9,10]. The present experiments connect these two aspects by varying the specimen orientation while maintaining the same prescribed LUU protocol. The observed orientation-dependent differences therefore indicate that, for the tested clay, characterization of the unloading stress path alone does not fully define the macroscopic response; the material orientation relative to the applied principal-stress axes is also relevant.

The results also indicate that different comparisons within the test matrix should not be assigned the same interpretive weight. In particular, the higher fitted initial tangent modulus obtained from the LUU tests relative to the CCU tests is protocol-dependent because the two test series employed different control modes and loading rates. Consequently, the within-path comparisons among different orientation angles, for which the testing protocol remains unchanged, provide a more direct basis for evaluating orientation dependence than the absolute stiffness difference between CCU and LUU. Similarly, the apparent *c* and *φ* values derived from two stress states are most appropriately used as comparative descriptors of stress-path and orientation-dependent strength response over the investigated pressure range rather than as unique material constants. These distinctions are also relevant when comparing the present results with previous unloading studies, which involve different soils, loading histories, drainage conditions, and unloading protocols [11,12,13,14,15]; direct numerical agreement is therefore not necessarily expected.

From a constitutive perspective, the present results support retaining the hyperbolic Duncan–Chang framework while modifying its parameterization for the prescribed LUU condition. The adjusted coefficients of determination for the linearized hyperbolic relationships range from 0.908 to 0.999, indicating that the underlying hyperbolic representation remains generally applicable across the investigated orientations and stress levels. The contribution of the present extension is therefore not to replace the Duncan–Chang framework, but to incorporate the experimentally observed orientation dependence into its LUU tangent-modulus formulation through orientation-conditioned parameters. The internal orientation hold-out check further shows that these parameter–angle relationships can interpolate an uncalibrated intermediate orientation within the present test matrix. This result should be interpreted as support for the internal consistency and interpolation capability of the parameterization, rather than as independent validation of complete stress–strain or field-scale performance. Accordingly, the proposed formulation is best regarded as an empirical fixed-orientation extension for the investigated LUU condition, providing a basis for subsequent evaluation under broader material and boundary-value conditions.

## 6. Conclusions

(1) Triaxial tests were conducted to investigate the effects of the initial major principal stress direction angle (α) and stress path on the mechanical behavior of remolded clay. The recorded responses were strain-hardening within the measured ranges; no objective yield point was determined, and no distinct peak was identified. Under the CCU path, the stress level adopted as the failure state generally decreases as α increases from 0° to 60° and then increases at α = 90°, with its maximum and minimum occurring at α = 0° and 60°, respectively. Under the LUU path, the measured terminal deviator stress exhibits a pressure-dependent orientation trend: at 100 kPa, the minimum occurs at α = 45°, followed by an increase at α = 60° and a subsequent decrease at α = 90°; at 200 kPa, it decreases monotonically from α = 0° to 90°. The initial tangent modulus decreases with α under both test protocols.
(2) The two-state Mohr–Coulomb indices exhibit clear differences between the tested stress paths and orientations. Under the CCU path, the apparent cohesion generally increases with α, whereas the apparent friction angle decreases overall. Under the LUU path, the apparent friction angle decreases with α, while the apparent cohesion decreases to a minimum at α = 45° and then increases. Because these indices were derived from only two adopted stress states at each orientation, they should be interpreted as comparative values over the tested 100–200 kPa range rather than as uniquely validated material constants.
(3) An orientation-dependent Duncan–Chang extension was established for the prescribed LUU path. The modulus number *K* decreases monotonically with increasing α, consistent with the reduction in fitted initial stiffness. The pressure exponent *n* shows an overall increase with α but retains local non-monotonic variations between adjacent orientations, reflecting the orientation dependence of the pressure sensitivity over the tested 100–200 kPa range. The parameter *R_f_* is retained as an orientation-specific stress-level mapping factor and exhibits a shallow U-shaped variation, with its minimum near α = 45–60°. These α-dependent parameters are incorporated into the orientation-dependent tangent-modulus expression to represent the fixed-orientation unloading response. An internal orientation hold-out check, with α = 60° excluded from calibration, produced absolute relative deviations of 2.1% or less for the fitted orientation-dependent parameters and 2.9% for the derived initial tangent modulus at the two tested pressures. The result supports interpolation within the investigated angular range. The principal constitutive contribution of this study is therefore the explicit linkage of the initial material orientation to the parameterization of the lateral unloading response within the Duncan–Chang framework.
(4) At the material and constitutive-modeling level, the present results provide a reference for researchers and geotechnical analysts concerned with stress-path and orientation-dependent clay behavior. The proposed formulation is currently limited to the tested remolded clay, consolidation pressures of 100 and 200 kPa, the prescribed saturated LUU path, and the investigated orientation range of 0–90°. Future work should evaluate the model through boundary-value simulations of supported excavations and extend its applicability to other soils, stress levels, and loading conditions. Broader extensions may further consider coupled environmental effects, such as thermal exposure [30], within multiphysics analyses.

## Figures and Tables

**Table 1:** Basic index properties of the tested clay.

Property	Symbol	Value
Water content	ω	28.0%
Bulk density	*ρ*	1.79 g/cm^3^
Liquid limit	*W_L_*	41.8%
Plastic limit	*W_P_*	25.9%
Plasticity index	*I_P_*	15.9%

**Table 2:** Testing program.

Test Scheme	Isotropic Consolidation	α (Angle Between Sampling Direction and Deposition Direction)	Shearing Process (Undrained)	Control Mode	Shearing Rate
CCU	100 kPa	0°, 30°, 45°, 60°, 90°	${\Delta \sigma }_{1}>0$	Strain control	0.05%/min
	200 kPa	0°, 30°, 45°, 60°, 90°	${\Delta \sigma }_{3}=0$		
LUU	100 kPa	0°, 30°, 45°, 60°, 90°	${\Delta \sigma }_{1}=0$	Stress control	0.2 kPa/min
	200 kPa	0°, 30°, 45°, 60°, 90°	${\Delta \sigma }_{3}<0$		

**Table 3:** Shear strength parameters under different stress paths.

Test Scheme	Initial Orientation of Principal Stress (°)	Cohesion *c* (kPa)	Internal Friction Angle φ (°)
CCU	0	26.3	21.6
	30	27.6	19.3
	45	29.6	18.6
	60	35.3	14.4
	90	38.7	15.1
LUU	0	19.1	16.9
	30	18	16.1
	45	16.3	15.1
	60	17.2	14.7
	90	18.5	14.6

**Table 4:** Adjusted coefficients of determination ($R_{\mathrm{adj}}^{2}$) for the linearized hyperbolic fits.

α (°)	CCU, 100 kPa	CCU, 200 kPa	LUU, 100 kPa	LUU, 200 kPa
0	0.997	0.997	0.998	0.993
30	0.987	0.988	0.974	0.953
45	0.997	0.998	0.998	0.998
60	0.998	0.995	0.999	0.938
90	0.949	0.993	0.908	0.983

**Table 5:** Fixed-orientation quantities used for the LUU Duncan–Chang extension.

Initial Orientation of Principal Stress α (°)	$p_{c}^{\prime }$ (kPa)	$q_{ult}$ (kPa)	$E_{i}$ (kPa)	*c* (kPa)	φ (°)	*K*	*n*	$R_{f}$
0	100	171	17,391	19.1	16.9	173.91	0.470	0.853
	200	272	24,096					
30	100	185	15,848	18	16.1	158.48	0.447	0.848
	200	207	21,598					
45	100	145	10,341	16.3	15.1	103.41	0.814	0.844
	200	206	18,182					
60	100	141	10,081	17.2	14.7	100.81	0.772	0.843
	200	197	17,212					
90	100	127	7117	18.5	14.6	71.17	1.001	0.848
	200	186	14,245					

**Table 6:** Internal orientation hold-out check with α = 60° excluded from calibration.

Parameter	Predicted at 60°	Reference at 60°	Absolute Relative Deviation (%)
*c* (kPa)	17.01	17.20	1.1
*φ* (°)	15.01	14.70	2.1
*K*	102.74	100.81	1.9
*n*	0.786	0.772	1.8
*R_f_*	0.8449	0.8430	0.2
*E_i_*, 100 kPa (kPa)	10,274	10,081	1.9
*E_i_*, 200 kPa (kPa)	17,714	17,212	2.9

## Data Availability

The original contributions presented in this study are included in the article. Further inquiries can be directed to the corresponding author.
